# Apicidin biosynthesis is linked to accessory chromosomes in *Fusarium poae* isolates

**DOI:** 10.1186/s12864-021-07617-y

**Published:** 2021-08-04

**Authors:** Thomas E. Witte, Linda J. Harris, Hai D. T. Nguyen, Anne Hermans, Anne Johnston, Amanda Sproule, Jeremy R. Dettman, Christopher N. Boddy, David P. Overy

**Affiliations:** 1 Agriculture and Agri-Food Canada, Ottawa Research and Development Centre, Ottawa, Canada; 2 Department of Chemistry and Biomolecular Sciences, University of Ottawa, Ottawa, Canada

**Keywords:** *Fusarium poae*, Metabolomics, Genomics, Secondary metabolites, Apicidin, Fungal plant pathogens, Mass spectrometry, Accessory chromosomes, Biosynthetic gene clusters

## Abstract

**Background:**

Fusarium head blight is a disease of global concern that reduces crop yields and renders grains unfit for consumption due to mycotoxin contamination. *Fusarium poae* is frequently associated with cereal crops showing symptoms of Fusarium head blight. While previous studies have shown *F. poae* isolates produce a range of known mycotoxins, including type A and B trichothecenes, fusarins and beauvericin, genomic analysis suggests that this species may have lineage-specific accessory chromosomes with secondary metabolite biosynthetic gene clusters awaiting description.

**Methods:**

We examined the biosynthetic potential of 38 *F. poae* isolates from Eastern Canada using a combination of long-read and short-read genome sequencing and untargeted, high resolution mass spectrometry metabolome analysis of extracts from isolates cultured in multiple media conditions.

**Results:**

A high-quality assembly of isolate DAOMC 252244 (*Fp*157) contained four core chromosomes as well as seven additional contigs with traits associated with accessory chromosomes. One of the predicted accessory contigs harbours a functional biosynthetic gene cluster containing homologs of all genes associated with the production of apicidins. Metabolomic and genomic analyses confirm apicidins are produced in 4 of the 38 isolates investigated and genomic PCR screening detected the apicidin synthetase gene *APS1* in approximately 7% of Eastern Canadian isolates surveyed.

**Conclusions:**

Apicidin biosynthesis is linked to isolate-specific putative accessory chromosomes in *F. poae*. The data produced here are an important resource for furthering our understanding of accessory chromosome evolution and the biosynthetic potential of *F. poae*.

**Supplementary Information:**

The online version contains supplementary material available at 10.1186/s12864-021-07617-y.

## Background

Fusarium head blight (FHB) is an economically devastating cereal crop disease that reduces yields, contaminates grains with harmful mycotoxins, and represents a threat to global food security [1]. Surveys of FHB-symptomatic cereals often detect a complex of different *Fusarium* species, including *F. graminearum, F. avenaceum* and *F. poae* [2–5]. While epidemic-level outbreaks of the disease on wheat and barley are attributed to aggressive isolates of *F. graminearum*, the routine detection of other close relatives in the *F. sambucinum* and *F. incarnatum-equisiti* species complexes in FHB-infected plants implies an intricate disease model involving fungi with varying levels of pathogenicity. In a recent survey of Canadian wheat, barley and oats, *F. poae* was the *Fusarium* species most frequently isolated from FHB-symptomatic oat samples and was as frequently isolated from barley as was *F. graminearum* [4]. The association of *F. poae* with FHB-damaged cereals is a global trend [5–7] that calls for investigation into fundamental aspects of the species’ life cycle, population structure and biosynthetic potential.

*F. poae* can produce a diverse range of mycotoxins including a mixture of type A and type B trichothecenes [8]. Neosolaniol, diacetoxyscirpenol, monoacetoxyscirpenol, fusarenone-X, nivalenol and scirpentriol have all been detected from *F. poae* isolates [9], and the extent of their accumulation in cereal grains depends on the producing isolate, the grain type and the environmental context in which the plants are grown [10]. Trichothecene detection and characterization are a top priority for food safety analysts due to their deleterious effects as protein synthesis inhibitors and their alteration of membrane properties [11]. However, the toxigenic potential of *F. poae* isn’t limited to trichothecenes. Beauvericin, enniatins and fusarins have also been detected from *F. poae* isolates grown in laboratory conditions and from FHB-damaged grain [9]. While none of these mycotoxins are monitored or regulated in Canada or Europe, in part due to insufficient studies of in vivo toxicity and lack of toxicokinetic data [12, 13], in vitro and in vivo studies suggest many of these molecules have genotoxic effects, have immunomodulating activity, and in some cases pose a reproductive health hazard to consumers [13]. Molecules such as enniatin and beauvericin have been labeled ‘emerging mycotoxins’ and may be placed under increased regulatory scrutiny in the future due to our evolving understanding of their in vivo bioactivity. Furthermore, the potential roles of these molecules (often termed ‘secondary metabolites’) in the context of plant invasion are poorly understood.

Technological advances in analytical chemistry now enable untargeted, simultaneous detection and identity prediction of complex secondary metabolite mixtures, providing an unprecedented view of fungal biosynthetic output. When coupled with high quality long-read genome sequencing tools, untargeted chemical profiling can be used to correlate metabolomes of individual fungal isolates to biosynthetic gene cluster (BGC) diversity within populations. BGCs are usually defined by their core, molecular scaffold-building enzymes, which include polyketide synthases (PKS), non-ribosomal peptide synthetases (NRPS), or terpene synthase/cyclases. Predicted clusters also include ‘tailoring’ enzymes such as oxidoreductases and methyl transferases, as well as transcription factors and transport-related genes. Genes within BGCs are presumed to be co-regulated during activation of the cluster – however the environmental or biological triggers for BGC activation are multifactorial. Pan-genomic analyses indicate *Fusarium* species, including *F. poae,* possess many undescribed ‘cryptic’ BGCs which may not be expressed under common in vitro culturing conditions [14–16]. The secondary metabolite products of these ‘cryptic’ BGCs may play critical roles in other contexts and may provide important clues as to how pathogens evolve. Developing a more complete picture of *F. poae* BGCs and associated secondary metabolites is therefore a key step in advancing our understanding of *F. poae* pathogenicity, particularly since many fungal plant pathogen genomes are dynamic and show the potential for rapid adaptation in response to plant defense mechanisms.

Profiling *F. poae* populations using untargeted metabolomics and long-read genomics is also prudent due to the recent discovery of accessory chromosomes (ACs) with secondary metabolite biosynthetic potential residing in a European isolate of the fungus [17]. ACs can be differentiated from core chromosomes by their non-Mendelian inheritance patterns, low gene density, high transposable element (TE) content and gene duplication frequencies. ACs have been studied extensively in other species of pathogenic fungi including *Alternaria alternata* [18], *F. solani* (formerly also known as *Nectria haematococca*) [19] and *F. oxysporum f. sp. lycopersici* [20]. In specific contexts relating to plant pathogenicity, ACs have been termed ‘pathogenicity chromosomes’ or ‘conditionally dispensable chromosomes’. Although we recognize the use of the term AC departs from the equally valid term ‘supernumerary chromosome’ (previously used in conjunction with *F. poae*) [17]*,* we believe that use of the term AC is more broadly consistent with fungal research literature as it relates ACs to ‘accessory regions’ (that share many of the key characteristics of ACs but reside on core chromosomes) [21]. ACs have been associated with novel plant host invasion in cases where they harbour fungal virulence factors (such as host specific toxins and effector proteins) and play an important role in plant pathogen niche invasion and adaptation [22]. The origins of ACs and associated genes are not always clear and may be diverse, including horizontal transfer between species/isolates or duplications and losses of core chromosome segments [23]. AC genetic content and the effects of disruptive TE transpositions between ACs and core chromosomes may generate novel genotypes in *F. poae* populations [17], promoting the evolution of increased virulence or niche invasion. Furthermore, *F. poae* isolates could theoretically produce AC-associated mycotoxins not currently screened by regulatory agencies in addition to the mycotoxins they are known to produce.

Previously published field surveys characterizing the occurrence of FHB in Eastern Canada from 2006 to 2017, found *F. poae* to be associated with symptomatic wheat, barley and oat heads [4]. Herein, from a sample set of 184 Eastern Canadian isolates of *F. poae*, a subset of 38 isolates were chemically profiled using an untargeted UPLC-HRMS based metabolomics analysis, and the results compared to genome sequences in a ‘multi-omics’ approach focused on secondary metabolite detection, AC prediction and BGC analysis. This approach permits the correlation of predicted isolate-specific ACs encoding BGCs with chemical profile patterns. Finally, we present a high-quality genome assembly for isolate *Fp*157 which includes seven predicted accessory chromosome contigs totalling 5.2 Mb or 8% of the total genome predicted size. This is a valuable resource to develop a greater understanding of the biosynthetic potential and structure of ACs in *Fusarium*.

## Results

### Isolate selection for genomic and metabolomic analysis

All isolates under investigation were initially identified morphologically and then confirmed as *F. poae* by *TEF1-α* gene sequence homology with other sequenced Fusaria at the Fusarium-ID website (http://isolate.fusariumdb.org/blast.php) [24]. From a culture collection of 184 *F. poae* isolates, a subset of 38 isolates were selected for detailed genomic and metabolomic analysis (Additional file 1 contains a list of all isolates screened). The selection of the 38 isolates was based on: genetic variance associated with *TEF1-α* and trichothecene biosynthetic genes *TRI1* and *TRI8* (inferred phylogenetic tree is in Additional file 2); diverse metabolomic signatures from ultra-high performance liquid chromatography coupled to high resolution mass spectrometry (UPLC-HRMS) data of extracts from isolates grown on YES media; and variation in host crop and geographical origin. *TRI1* and *TRI8* were chosen due to previous analyses which showed variations in these genes led to alternate trichothecene modifications [25–27] and higher sequence divergence observed within previously genome sequenced *F. poae* isolates [17].

Genome assembly using Illumina sequence data of the 38 chosen isolates produced a median of 1375 scaffolds per genome and a mean genome length of 39.25 Mb. A summary of Illumina genome assembly statistics can be found in Additional file 3. To evaluate the completeness of the assemblies we identified BUSCO (Benchmarked Universal Single Copy Orthologs) [28] gene analysis using the Hypocreales_odb10 database, which revealed all genomes were over 97% complete, with the exception of one poor quality genome (*Fp*030; 80.7%), and one failed sequencing run (*Fp*029, not included in genomic analysis).

### A high-quality genome for *Fp*157 confirmed the secondary metabolite biosynthetic potential in *F. poae* and the presence of accessory chromosome-associated sequences

**Fig. 1:**
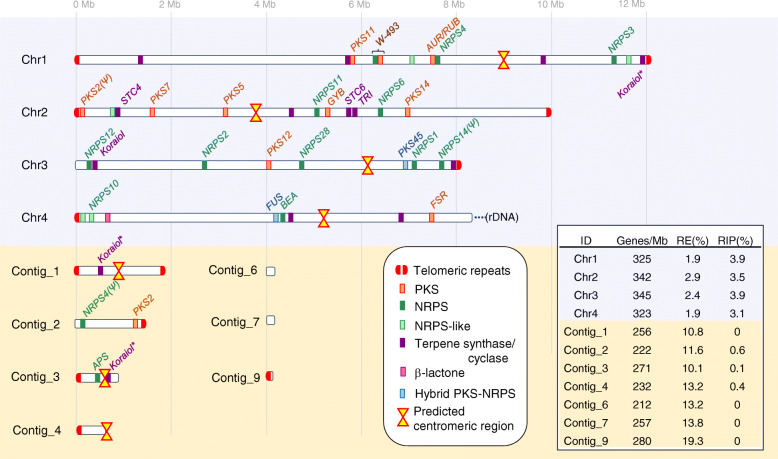
Genome assembly of *Fusarium poae* isolate *Fp*157 with predicted biosynthetic gene clusters (BGCs), centromeric regions and telomeric repeats overlaid. BGC sizes are not to scale. Asterisks indicate duplicated koraiol synthases with > 98% nt ID. Annotations above BGCs refer to associated synthase/synthetase clades, or associated mycotoxin products where known. RE indicates repeat element contents expressed as a percentage of each chromosome or contig length calculated independently of the rest of the genome (repeat content attributable to duplications between chromosomes was not calculated). Similarly, evidence of repeat-induced point mutation (RIP) was calculated independently per sequence and is expressed as the percent of each sequence predicted to be RIP-affected (evidenced by calculation of low GC-content compared to average dinucleotide frequencies). Predicted centromeres and telomeres were removed from all sequences prior to RIP analysis

*F. poae* isolate *Fp*157 was selected for long-read sequencing using the Oxford Nanopore platform (340,123 filtered reads, mean length of 21,465 bp and mean qscore of 10.24). We generated a high quality genome (Fig. 1) with four core chromosomes exhibiting strong macrosynteny to the previously genome sequenced Belgian *F. poae* isolate 2516 [17] as well as *F. graminearum* isolate PH1 [29]. Additional file 4 contains genome assembly statistics for *Fp*157, and Additional file 5 contains LASTZ [30] dot plots comparing core chromosome synteny between *Fp*157, *F. poae* 2516 and *F. graminearum* PH1. A total of 14,114 genes were predicted, with two additional genes manually annotated based on blastn matches to biosynthetic genes (*FpPKS2* and *FpNRPS4*, discussed in text). Core chromosomes Chr1 and Chr2 represent ‘telomere to telomere’ sequences with putative centromeric regions and telomeres comparable to the length, position and GC content of those described from *F. graminearum* PH1 [29]. Centromeres consist of approximately 50Kb-long regions averaging 15% GC content, and telomeres show canonical ‘TTAGGG’ repeats followed by a 1500 bp region averaging 37% GC content. Chr4 has telomeric repeats at the 5′ end, whereas the 3′ end encodes predicted rDNA repeats, also congruent with *F. graminearum* PH1. Chr3 is missing a telomeric sequence at the 5′ end and when compared to isolate 2516, subtelomeric regions appear inverted and rearranged. Lastly, mapping of the raw Nanopore and Illumina reads (data not shown) did not support the presence in *Fp*157 of the approximately 1 Mb inversion detected in Chr1 of isolate 2516. This inversion is also not observed in Chr1 of *F. graminearum* PH1 (See Additional file 5 for LASTZ comparisons) [17].

In addition to the core chromosomes, 9 contigs were assembled in the *Fp*157 genome ranging in size from 100,057 bp to 1,877,593 bp. Contig_5 is 140,862 bp long, representing the mitochondrial genome, and was not annotated. Contig_8 is 122,757 bp of rDNA repeats and is virtually identical to the 60Kb of rDNA repeats associated with the 3′ end of Chr4. The remaining seven contigs cumulatively total 5,205,433 bp and are presumed to represent AC sequences based on a number of factors. First, predicted ACs have genetic content that is congruent with the published content of predicted ACs in Belgian *F. poae* isolate 2516 [17]. Second, there is a lack of macrosynteny to sister species *F. graminearum* PH1 core chromosome sequences. Third, contigs are predicted to be less affected by repeat-induced point mutations (RIP, predicted by sliding-window dinucleotide frequency analysis [31]), when compared to core chromosomes, a pattern also congruent with *F. poae* 2516 ACs. Fourth, the contigs show elevated repetitive element content compared to core chromosomes, as seen in many confirmed fungal ACs [32]. Finally, the contigs show lower predicted gene densities when compared to core chromosomes, another characteristic feature of ACs [20, 33] (Fig. 1).

Within the AC-associated contigs, there are three putative centromeric regions identified by similar size and GC content to core centromeres (contig_1, 52.7 kb averaging 14.5% GC content; contig_3, 53.3 kb averaging 14.7% GC content; contig_4, 35.2 kb terminating at end of contig, averaging 15.6% GC content). Additionally, six sets of telomeric repeats were identified and are all located on contig terminal regions. We therefore suggest there are three ACs in total, however further experimental verification including electrophoretic karyotyping is needed to confirm the number and size of the ACs, and to build telomere-to-telomere assemblies of their contents.

AntiSMASH 5.1 [34] analysis of the *Fp*157 genome predicted 43 discrete BGCs which encoded various core scaffold genes including 12 polyketide synthases (PKS), 13 terpene synthases, 10 non-ribosomal peptide synthetases (NRPS), 10 NRPS-like synthetases (usually a single NRPS module lacking a canonical domain) and 2 hybrid PKS-NRPS genes (Table 1). One cluster was predicted to be involved in the formation of β-lactones. Blastx comparison of NRPS adenylation domains, PKS ketosynthase domains and full-length PKS genes (all domains) to published databases of *Fusarium*-associated PKS and NRPS genes [15, 35] indicated all PKS and NRPS genes are orthologs of genes previously associated with Fusaria, and approximately half are associated with known products.

**Table 1:**
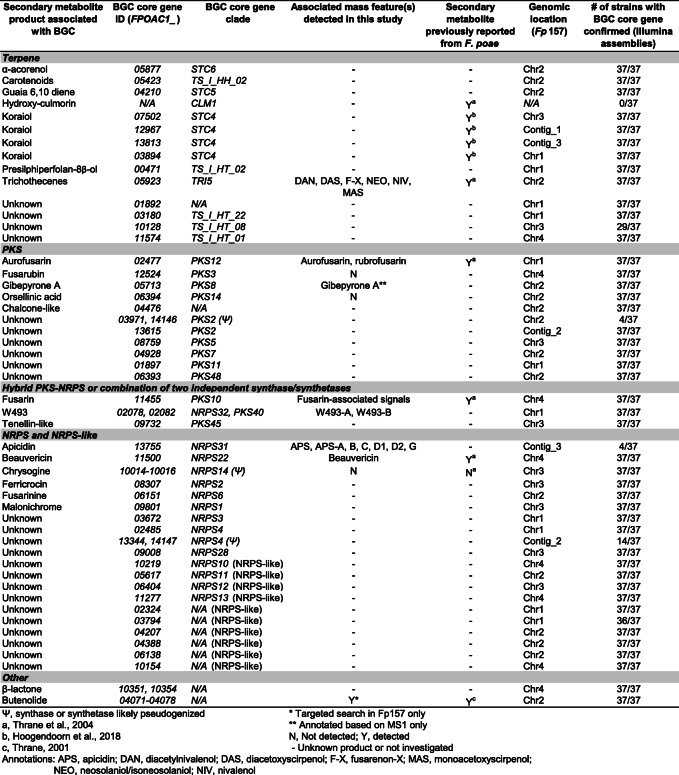
Summary of predicted BGCs from the *Fp157* genome and associated mass features detected in this study

### Chemical phenotyping of *F. poae* isolates

**Fig. 2:**
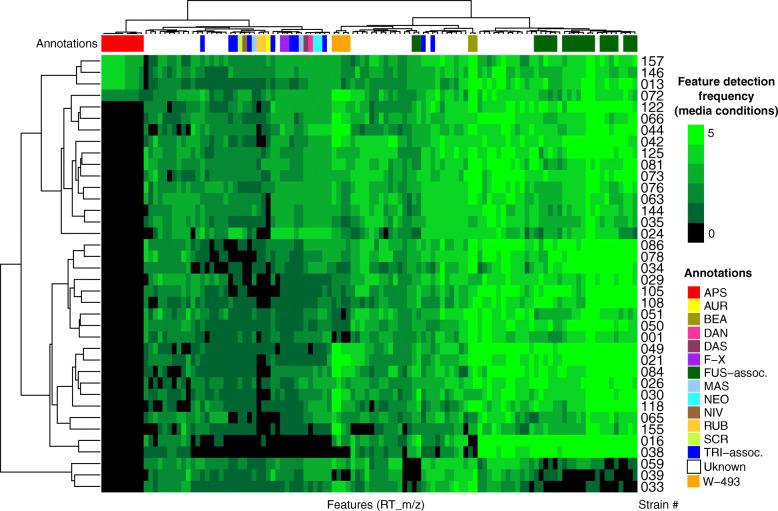
Consensus chemical phenotypes of 38 Canadian isolates of *Fusarium poae*. Heatmap values represent the frequency of detection for mass features averaged over five media conditions per isolate. Dendrograms at left and top generated from hierarchical cluster analysis of detection frequencies. Annotations: APS, apicidins and apicidin-like features; AUR, aurofusarin; BEA, beauvericin; DAN, diacetylnivalenol; DAS, diacetoxyscirpenol; F-X, fusarenon-X; FUS-assoc., fusarin-associated features; MAS, monoacetoxyscirpenol; NEO, neosolaniol/isoneosolaniol; NIV, nivalenol; RUB, rubrofusarin; SCR, scirpentriol; TRI-assoc., trichothecene-associated features other than those verified by comparison to commercial standards or published MS^2^ spectra (see text); W-493, cyclodepsipeptides W-493 A/B

Chemical phenotypes for each of the 38 isolates grown in vitro were generated to visualize patterns in mass feature detection frequencies, and untargeted mass feature diversity. Mass features were obtained from UPLC-HRMS profiles of extracts from isolates grown on five media conditions. Each media condition was chosen to diversify sources of nitrogen, sugars, salt stress and starvation stress. Media formulations used are detailed in Additional file 6. Mass feature intensities were converted to binary presence/absence for each media condition, and then averaged across all media conditions into a consensus phenotype for each isolate. Figure 2 represents consensus chemical phenotypes from all isolates and includes all mass features discussed in this study as well as a subset of unannotated signals (see Additional file 7 for expanded analysis).

Hierarchical cluster analysis grouped the consensus chemical phenotypes (‘metabolomes’) by metabolite detection pattern similarities between isolates and between mass features (Fig. 2, left and top dendrograms). Mass features annotated as trichothecenes and fusarins were present in large clusters due to the many functional alterations present in these molecular families. Close examination of raw MS data from isolates *Fp*016 and *Fp*038 revealed extracts from all media conditions were dominated by the relative abundance of fusarin-associated signals. This likely led to a skewed mass feature profile (with many mass features falling below the limit of detection) due to sample dilution prior to injection, and possibly again during data preprocessing, which could explain the absence of beauvericin and some trichothecene-associated signals from these isolates. Fusarins made up a significant portion of signals from nearly all isolates on all media types, with the exception of isolates *Fp*059, *Fp*039 and *Fp*033 which had lower frequencies of fusarin-associated signals relative to the other isolates. We concluded that isolates exhibited varying levels of core-chromosome associated signals. Furthermore, we noted four isolates, *Fp*157, *Fp*013, *Fp*088 and *Fp*072, exhibited mass features which were absent from all other isolate profiles, and were therefore predicted to be AC-associated based on the presumption that AC biosynthetic content varies across populations of *F. poae*. These mass features were annotated as apicidins.

### Annotation of type A and B trichothecenes

Comparison of trichothecene-associated signals to commercial standards confirmed the presence of both types A and B which have been previously associated with *F. poae.* Isolates grown on YES media appeared to produce the most trichothecene-associated mass features, a pattern which is unsurprising since this media is rich in sucrose, a known trigger for trichothecene production in the closely-related species *F. graminearum* [36]. Mass features associated with 15-diacetoxyscirpenol, 15-monoacetoxyscirpenol, neosolaniol and fusarenon-X were confirmed by comparison to standards. A feature with the same *m/z* and similar fragmentation pattern as neosolaniol was detected, eluting slightly later than neosolaniol, and is therefore suggested to be iso-neosolaniol (either 4,8 diacetyl or 8,15 diacetyl form). Additionally, mass features were annotated as diacetylnivalenol and scirpentriol based on in silico fragmentation comparison. A mass feature matching a commercial nivalenol standard was detected from most isolates but was a low-intensity signal compared to other trichothecenes. Other signals associated with trichothecene biosynthesis matched *m/z* and chemical formulas of trichothecene precursors such as isotrichotriol, or analogs of the major type A and B trichothecenes detailed above, and were tentatively annotated as “trichothecene-associated” due to the absence of publicly available MS^2^ spectra or commercial standards at this time.

3A-deoxynivalenol, 15A-deoxynivalenol, T-2, HT-2 and T-2 tetraol- associated *m/z* were not detected from any isolate grown in this study. To test whether isolates had the genetic capability to produce T-2 or HT-2 toxin, we surveyed (by blastp) the 37 *F. poae* Illumina genomes using the acetyl transferase TRI16 from *F. sporotrichioides*, shown to facilitate esterification of the C-8 hydroxyl group in trichothecenes during production of T-2 toxin [37], and no *TRI16* homologs were detected.

### Confirmation of aurofusarin, beauvericin, fusarin, W-493 and other metabolites

Next, we annotated mass features associated with non-trichothecene mycotoxins. Beauvericin was confirmed by comparison to a commercial standard. Cyclodepsipeptides W-493 A and W-493 B [38] were annotated by comparison to GNPS-supplied MS^2^ fragmentation spectra (Mirror plots comparing MS^2^ spectra are in Additional file 8). Fusarin-associated signals were numerous, likely owing to the multiple stereoisomers commonly observed from fusarin-producers [39, 40]. Features matching the *m/*z, predicted in silico fragmentation patterns and UV spectra of aurofusarin and its precursor rubrofusarin were detected from most isolates but production was not consistent to isolate groupings or media conditions. A mass feature matching *m/z* of gibepyrone A was detected, but could not be confirmed due to lack of standards and experimentally derived MS^2^ spectral database representation, and *in-silico* MS^2^ spectra generation was inconclusive. Additionally, we searched for genomic and metabolomic evidence for production of butenolide, a cytotoxic secondary metabolite reported from F. poae [41]. A gene cluster homologous to the butenolide-associated cluster in *F. graminearum* [42] was detected in the *Fp*157 genome. We reasoned that due to the highly polar nature of butenolide, it likely eluted with the injection peak during chromatography and was therefore excluded from our UPLC-HRMS chemical phenotypes (the injection peak was sent to waste to prevent soiling of the MS inlet due to media components). Extracts from *Fp*157 were re-profiled by UPLC-HRMS without diverting the injection peak, and butenolide production was confirmed in this isolate (see Additional file 9 for details).

Several secondary metabolites predicted from the antiSMASH analysis for *F. poae* (Fp157) were not detected. Notable absences include fusarubin (and other analogs associated with the *fsr1* or *PKS3* cluster [43]), orsellinic acid and chrysogine. Fusarubin and orsellinic acid analogs may be among the unannotated signals here and will be the subject of further investigation. Closer inspection of the chrysogine-associated *NRPS14* homolog in *F. poae* isolates indicates it has been disrupted by an 800 bp insertion of very low GC content (< 10%) in all isolates including the Belgian *F. poae* isolate 2516. Although hydroxy-culmorins have been previously detected from *F. poae* isolates [9], we were unable to find homologs of the longiborneol synthase gene *CLM1,* shown to be required for culmorin production in *F. graminearum* [44]*,* in any of the *F. poae* assemblies generated in this study. Hydroxy-culmorin-associated mass features were detected in the metabolomes of nearly all isolates (except *Fp*016), but lack support due to the absence of available commercial standards. The presence of hydroxy-culmorins is thus insufficiently supported at this time to warrant annotation.

Taken together, our data confirms Eastern Canadian *F. poae* isolates produce similar chemical profiles to European *F. poae* isolates when grown in vitro, as pertaining to trichothecene, fusarin, beauvericin and aurofusarin production. Our untargeted analysis of chemical profiles detected cyclodepsipeptides W-493-A and B associated signals, and highlighted mass features not matched in our database of known *Fusarium* mycotoxins which could represent undescribed secondary metabolites (Fig. 2).

### The production of apicidin and its derivatives is linked to the accessory chromosome in *Fp*157

**Fig. 3:**
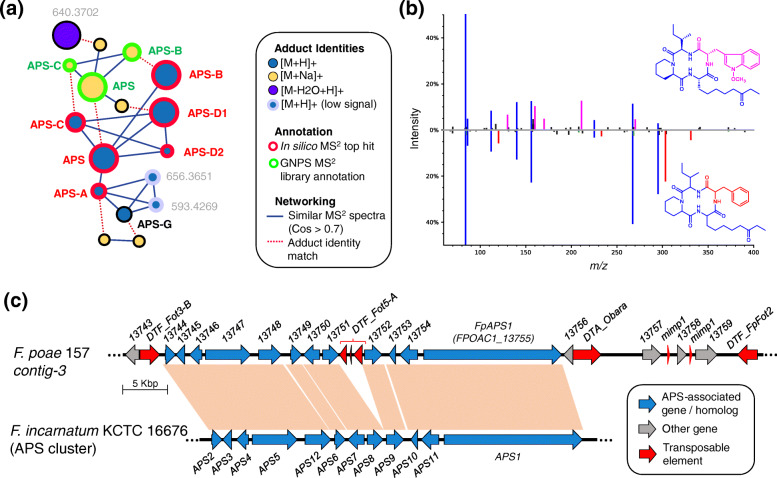
(**a**) Apicidin (APS) subnetwork generated from feature-based molecular network analysis of APS-like signals using GNPS (release_23) [47], visualized in cytoscape. Nodes represent distinct features (peaks) with unique retention times and *m/z*, and are either connected by cosine similarity score (threshold = 0.7, blue line) or adduct identity match generated using IIN module [48] in MZMine2 [49] (red line). Nodes are coloured based on ion identity, and node outlines are coloured by annotation method: red annotations derive from top hit from in silico MS^2^ structural prediction using Sirius / CSI Finger-ID [50], green annotations derive from spectral matching to GNPS database, grey outlines represent spectra whose adducts were annotated by manual inspection of raw data. Potentially novel APS-like signals are annotated with exact masses (< 5 ppm). Node size represents relative size of signal calculated by precursor intensity (sum of all spectra in MS^2^ scan). (**b**) Mirror plot comparing MS^2^ spectra of predicted APS and APS-G signals. Substructures are coloured based on association with *m/z* motifs: blue *m/z* occur in nearly all APS-associated mass feature MS^2^ scans, purple fragments are detected in most spectra associated with tryptophan-bearing apicidins, red fragments correspond to predicted phenylalanine moiety-associated fragments and appear only in putative APS-G spectra. For detailed information see Additional file 11. (**c**) Synteny visualization of *FpAPS* gene cluster residing on putative accessory chromosome of *Fp*157 as compared to homologous cluster in *F. incarnatum* KCTC 16676 (Genbank accession GQ331953) [51]. Blue arrows are predicted genes, red squares are predicted transposable elements. Predicted *APS* gene functions: 1, NRPS; 2, transcription factor; 3, pyrroline reductase; 4, aminotransferase; 5, fatty acid synthase; 6, O-methyl transferase; 7, cytochrome P450; 8, cytochrome P450; 9, FAD-dependent oxidase; 10, short-chain reductase; 11, efflux pump; 12, reductase

We investigated the detection of apicidins*.* Mass features were detected primarily from broth extracts of four of the 38 isolates cultured (*Fp*013*, Fp*072*, Fp*146*, Fp*157*)*, and included features with *m/z* and isotope ratios matching apicidin (APS) and APS analogs A, B, C, D1, D2, and G [45, 46]. To confirm these annotations, we used feature-based molecular network analysis and MS^2^ spectral matching (Fig. 3a). Network analysis showed all isolate-specific signals grouped into a single subnetwork. MS^2^ spectra from three mass features matched publicly available, experimentally-derived MS^2^ spectra from APS, APS B and APS C (mirror plots in Additional file 10). MS^2^ data from APS-A, D1, D2 and G, were not represented in experimentally-derived MS^2^ databases at the time of analysis. However, APS-A, D1 and D2 were supported by in silico predictions of potential fragmentation patterns from known molecular structures using Sirius / CSI Finger-ID. Lastly, APS G was annotated by manual examination of the MS^2^ spectra (Fig. 3b, see Additional file 11 for expanded analysis of APS G signals, and Additional file 12 for expanded network analysis of APS-associated signals). Molecular networking analysis indicated there were at least three unannotated signals which matched fragmentation patterns of the apicidin-like signals, suggesting they are novel APS analogs. The most intense of the three unknown APS-like signals had the same *m/z* as apicidin D1 [M + H]^+^ peak (*m/z* 640.3705) but was determined to be a [M-H_2_O + H]^+^ ion by the MZMine IIN module, with the [M + H]^+^ missing from the raw data, although a [M + Na]^+^ adduct signal was evident. The other two were detected at relatively low intensities and were not further investigated.

Genomic analysis of the *Fp*157 isolate revealed homologs of all 11 genes previously shown to be co-regulated during APS production [51], are present in a single, near-contiguous cluster located on AC-associated contig_3. We annotated this cluster as ‘*Fp*APS’. We compared the *Fp*APS cluster to the APS cluster annotated from Jin et al. 2010 using blastn (Fig. 3c) and determined all genes relating to APS production are found in the same relative order, with the exception of a DNA-transposon insertion between *APS8* and *APS9* in *Fp*157. This transposon matches *DTF_Fot5-A* from a previously published library of transposons described from the Belgian *F. poae* isolate 2516 [17], but appears to be fragmented (Fig. 3c).

Additionally, four genes with predicted roles in biosynthesis were found adjacent to the *Fp*APS cluster in *Fp*157 and are uniquely present in APS-producing isolate genomes. The co-localization of these genes to the *Fp*APS cluster indicates they may be involved in APS-associated molecular family diversification. *FPOAC1_13756* is a predicted amidase, which appears to have a *DTA_Obara* DNA transposon insertion (see [52] for explanation of TE nomenclature used here). This gene is also present in the homologous *APS* cluster in *F. sporotrichioides* and has not previously been associated with apicidin production. Three additional predicted biosynthetic genes are adjacent to the *Fp*APS cluster: *FPOAC1_13757* is an NRPS-like gene with an AMP-binding domain, *FPOAC1_13758* is an O-methyl transferase and *FPOAC1_13759* is an oxidoreductase with similarity to the ELFV-dehydratase family. Interestingly, when searched against the NCBI database, all three genes have top blastn hits in a three-gene cluster encoded by the strawberry anthracnose-causing mold, *Colletotrichum nymphaeae* isolate SA01, which is suggested to grow endophytically in weedy grasses [53]. Furthermore, the trio of genes are interspersed by two repetitive sequences with homology to ‘*miniature-impala’* or ‘mimp’ TEs which have been associated with pathogenicity genes on *Fusarium oxysporum* ACs [54]. The *Fp*APS cluster and adjacent genes are present in the genomes of all isolates from which APS-associated mass features were detected.

The possibility of the *Fp*APS cluster originating by horizontal gene transfer from another species was investigated by performing a Blastn analysis of the *Fp*APS genes against the NCBI nucleotide database. Top hits for all *Fp*APS genes matched coding sequences from close relatives *F. sporotrichioides* and *F. langsethiae*, with most at ~ 97% nucleotide identity, are provided in Additional file 13. As assemblies for *F. sporotrichioides* and *F. langsethiae* are not currently at chromosome-level, we cannot yet infer whether their APS-like BGCs are on ACs or core chromosomes. However, the location of the contig breaks in the *F. langsethiae* 201059 genome strongly suggests that it has the same transposable element (TE) insertion site between *APS8* and *APS9* and therefore this APS-like cluster is likely to be either on an AC or accessory region in this isolate.

Finally, to assess the frequency of occurrence of *FpAPS1* in our collection of 193 Ontario and Quebec *F. poae* isolates as well as 10 international isolates, we designed PCR primers which would specifically amplify a 150 bp nucleotide sequence from *F. poae APS1* and screened the genomic DNA (see Additional file 14 for representative sample gel lane). Of these, 15 isolates tested positive for the *APS1* gene, including an isolate collected from Ontario wheat in 2006 (DAOMC 239526), indicating the presence of the *FpAPS1* has persisted at least as long as the time period under study in Eastern Canada. See Additional file 1 for results of *APS1* screening. Active APS production was confirmed from 4 out of the 15 *FpAPS1* containing *F. poae* isolates (as they were the only ones included in the in-depth metabolomics analysis described above). None of the international isolates tested positive for the *APS1* gene.

## Discussion

In this study we have examined the secondary metabolite biosynthetic potential of 38 isolates of *F. poae* from Eastern Canada by analysis of genomes and chemical phenotypes. The combination of modern genome sequencing platforms and UPLC-HRMS profiling of fungal extracts provides a powerful approach for screening communities of fungal plant pathogens which may exhibit lineage-specific metabolite traits. In this case, untargeted chemical profiling enabled the confirmation of known mycotoxins associated with a ‘core’ *F. poae* chemical phenotype in addition to the discovery of an ‘accessory’-associated metabolome present only in a subset of isolates. These isolates are producing known and potentially novel forms of APS, a potent histone deacetylase inhibitor [55]. The high-quality genome of an APS-producing isolate, *Fp*157, indicates there are many biosynthetic gene clusters in this species which have not yet been associated with known products. Moreover, the presence of secondary metabolite BGCs on ACs can further diversify chemical phenotypes, underlining the desirability of untargeted metabolomic screening of population isolates to detect novel mycotoxin signatures.

Core chromosome-associated secondary metabolites described in this study generally agree with previously published data from European *F. poae* isolates cultured in vitro [56], with some minor exceptions. Production of the highly toxic T-2 and HT-2 toxins in grains infected with *F. poae* has been described [9, 57] but is not supported by recent genetic and chemotype analyses [56] including this study. The absence of *TRI16* in *F. poae* genomes generated here indicates Eastern Canadian isolates are unlikely to produce T-2 or HT-2 toxins regardless of the experimental conditions employed. Although it is possible that a *TRI16* homolog could reside on a isolate-specific AC or accessory region in other isolates, we believe it is also likely that previously reported *F. poae* T-2 and HT-2 producers were misidentified isolates of *F. langsethiae*, a known producer of T-2 and HT-2 with a very similar morphology to *F. poae*.

In addition to known mycotoxin confirmation, this study highlights undescribed biosynthetic potential in *F. poae* populations. From a genomics perspective, this includes roughly half the PKS clusters detected in *Fp*157, which have no predicted products (Table 1). Among these, PKS clades 7 and 8 are considered to be ubiquitous among all studied Fusaria, clade 5 is discontinuously distributed within *Fusarium*, and clades 45 and 48 are present in only a few Fusaria [35]. Similarly, products of some NRPS and NRPS-like clusters in *F. poae* are undescribed, including NRPS clades 3, 4 and 10–13 (all appear common among Fusaria [15]). Preliminary analysis suggests some of the unannotated metabolomics signals presented here may represent products of undescribed BGCs and provide targets for further molecular elucidation.

APS production and APS*-*associated BGCs have been detected from numerous *Fusarium* species at various levels of phylogenetic distance from *F. poae*, however the origins of this cluster in *F. poae* isolates remain unclear. APS was first detected from an isolate of the *Fusarium incarnatum-equiseti* species complex (FIESC) [58] and the gene cluster has since then been detected from *Fusarium* isolates across six species complexes [14, 59] including FIESC-12 (isolate NRRL 66336), which has since been reclassified as *Fusarium flagelliforme* [60], and FIESC-26 (isolate ATCC 74289 [58]), reclassified as *Fusarium hainanense* [60]. In vitro production of apicidins is confirmed from the FIESC [45, 58, 61], *F. langsethiae* [62], *F. fujikuroi* [63], and possibly *F. sambucinum* (isolates KCTC 16676 and 16677, identified by morphology only). The presence of APS-like clusters among diverse Fusaria suggests horizontal transfers of genes or ACs could be at play*.* However, blastn comparisons indicate the *Fp*APS genes share highest nucleotide identities to the closest known relatives of *F. poae*, including *F. sporotrichioides* and *F. langsethiae* (Additional file 13). This makes it challenging to predict whether the presence of *Fp*APS in *Fp*157 is the result of horizontal transfer from a close relative, or whether the cluster originates from a common ancestor and is retained by a small number of *F. poae* isolates*.* It is beyond the scope of this study to resolve this problem. More high quality long-read genomes will help unravel the evolutionary path of this BGC in *Fusarium.*

Although apicidins have demonstrated phytotoxicity towards wheat and maize [64], and were expressed by pathogenic *F. fujikuroi* isolates during growth in rice [65], their role during infection by plant pathogens, if any, is unknown. Apicidins have traditionally been recognized for their potent ability to inhibit histone deacetylase activity in apicomplexan parasites [55], and their use as antitumor therapeutics [66, 67]. Histone deacetylase inhibition can lead to hyper-acetylation of histones, impacting an organism’s ability to regulate genetic transcription. Apicidins are structurally similar to HC-toxin, another cyclic tetrapeptide histone deacetylase inhibitor with a well-documented role as a virulence factor during infection by the fungal plant pathogen *Cochliobolus carbonum* [68]. The recent detection of HC-toxin gene clusters in the genomes of *Alternaria brassicae* isolate J3 [69] (where it is assembled to a putative AC) and *Alternaria jesenskae* isolate AM237084 [70] suggests this cluster may have been horizontally transferred between species or even genera. As with the *FpAPS* cluster, the evolutionary origin of the HC-toxin cluster in *Alternaria* has not been ascertained. A recent study comparing chromosome counts found evidence for ACs in many FHB-associated APS producers, including *F. avenaceum, F. poae, F. sporotrichioides,* and members of the *F. incarnatum-equiseti* species complex [71]. Long-read genome sequencing of these Fusaria will cast light on whether the APS cluster is on an AC or accessory region in these species.

**Fig. 4:**
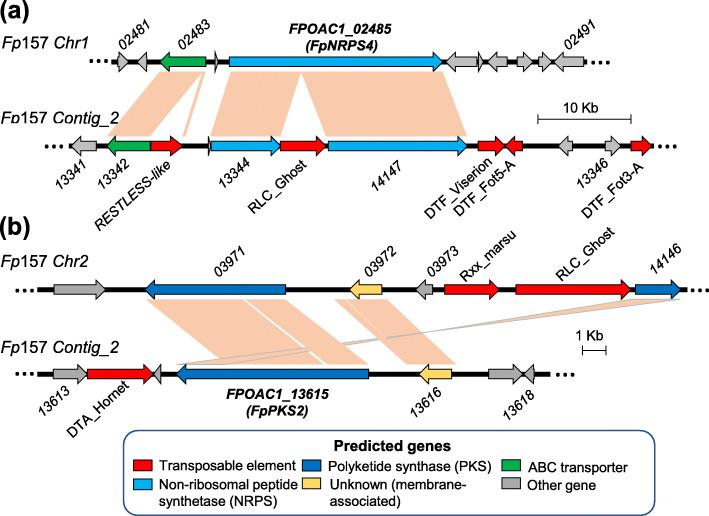
Gene synteny visualization of predicted BGCs on *Fp*157 core chromosomes and homologous regions on accessory chromosomes. (**a**) *FpNRPS4*-associated region on core chromosome 1 compared to homologous region on Contig_2. (**b**) *FpPKS2*-associated region on core chromosome 2 compared to homologous region on Contig_2

*Fp*APS was not the only secondary metabolite BGC identified on AC-associated contigs in *Fp*157. Paralogous genes from clades *NRPS4*, *PKS2* and *STC4* were represented on both core chromosome and AC-associated sequences, with paralogs sharing 70–80% nt identity (Table 1, Fig. 1). Although TE-disruption has likely pseudogenized the core chromosome-associated *PKS2* and AC-associated *NRPS4* paralogs in *Fp*157, their presence support the possibility of historical gene amplification and/or neofunctionalization (Fig. 4). It is unclear whether *FpPKS2* and/or the disrupted *FpNRPS4* located on predicted ACs originate from the duplication of core chromosome genes, interspecies hybridization (followed by chromosome/gene losses), or horizontal AC transfer from another species. By contrast, genomic evidence from *Fp*157 and Belgian *F. poae* isolate 2516 suggests gene duplication has occurred for *STC4* paralogs on ACs; three copies with greater than 98% nt ID were assembled in *Fp*157 and over six copies were assembled in *F. poae* 2516. Furthermore, the localization of one of the *STC4* copies to a subtelomeric region of a core chromosome in *Fp*157 underlines the potential for inter-chromosomal gene transfer of biosynthetic genes between ACs and core chromosomes in *F. poae*. Koraiol is the predicted product of *STC4* paralogs in *F. poae* 2516, and has been recently associated with pathogenic *F. fujikuroi* isolate growth *in planta* [65]*.* Clarifying the effects of koraiol synthase *in planta* will help generate testable hypotheses on the effects of its multiplication in *F. poae* genomes.

**Fig. 5:**
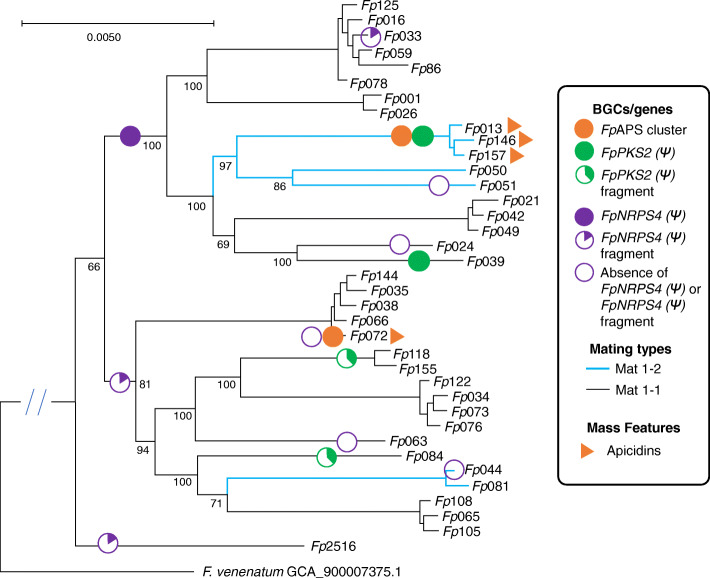
Phylogenetic analysis of 4141 single-copy BUSCO orthologs from *F. poae* isolates investigated in this study including Belgian isolate 2516*. F. venenatum* assembly GCA_900,007,375.1 was included as an outgroup to determine the true location of root (double slashes indicate truncated branch leading to outgroup). Evolutionary histories were inferred using the Maximum Likelihood (ML) method. Branch lengths are measured in number of substitutions per site, and node values indicate Ultrafast bootstrapping values (*n* = 1000). Biosynthetic gene annotations: the *Fp*APS cluster and the disrupted *FpNRPS4 (Ψ)* synthetase are associated with accessory chromosome sequences in the *Fp*157 assembly, whereas the disrupted *FpPKS2 (Ψ)* synthase was assembled to a core chromosome in *Fp*157. Purple and green pie charts represent size of fragments detected relative to concatenated *Fp*157 *FpNRPS4 (Ψ)* or *PKS2 (Ψ)* sequences*.* Empty pie charts indicate absence of *FpNRPS4 (Ψ)* detection

Mapping the various secondary metabolite clusters associated with ACs onto the *F. poae* BUSCO phylogeny presents a dynamic picture of AC-associated genes in *F. poae.* As seen in Fig. 5, the BUSCO-inferred clades divide into two groups based on widespread presence of *FpNRPS4 (Ψ)* paralog variants. In one group, isolates have complete *FpNRPS4 (Ψ)* representation (100% coverage when compared to *Fp*157), although in every instance *FpNRPS4 (Ψ)* is split between contigs, at the same site as the TE disruption in *Fp*157, implying the synthetase has been disrupted in all genomes in which it appears. The second group contains a truncated *FpNRPS4 (Ψ)* fragment with 15% coverage, suggesting the synthetase has further degraded in this lineage. Mating type MAT1–1 is the dominant mating type in this population, as was found in European populations [56]. Potential markers for ACs, including *FpPKS2, FpNRPS4 (Ψ)* fragments*, STC4* paralogs*,* and *Zit1* (a small TE previously associated with ACs in *F. poae* [72], data not shown), are detected in nearly all genomes, supporting the possibility of widespread ACs in *F. poae* populations.

The effects of AC-associated genes on *F. poae* isolate pathogenicity, if any, remain unexplored. Extensive profiling of *F. poae* populations is currently being undertaken in a Canada-wide survey to build on the work presented here, in combination with *in planta* pathogenicity trials to further explore the role of *F. poae* in the FHB disease complex. Critical issues remain outstanding with regards to *F. poae*, which would help shape hypotheses surrounding the utility of its secondary metabolite outputs: what is the complete life cycle of this fungus? What are its primary hosts or trophic states (pathogenic, saprotrophic or endophytic)? Are some isolates expanding their distribution at the expense of others?

*Fusarium poae* has been flagged as a potential danger to agriculture, and for good reason: the mycotoxins and emerging mycotoxins detected from this species have demonstrably harmful effects to living cells, disrupting key processes such as protein synthesis (trichothecenes), DNA transcriptional control (APS) and compartmentalization of ion gradients (beauvericin) [73]. Apicidin is not currently monitored in Canadian cereals and is not regulated anywhere globally. In a recent study of mycotoxin content in globally-sourced pig feeds, APS was detected in over half the feed samples and was found to be the most cytotoxic against pig gut endothelial cells in comparison with 27 other mycotoxins detected [74]. Less is known of the effects APSs might have on plant systems and the evolution of pathogenicity. For example, although the bioactivity of APS is well documented and likely not trivial to the plant infection process, there is as yet insufficient evidence to support the hypothesis that the presence of the *Fp*APS-bearing AC or any other AC associated with the isolates studied here improves the fitness of *F. poae* in the context of cereal crop invasion. Nevertheless, the potential ability of FHB-associated Fusaria to transfer and modify BGCs between species via ACs and other rapidly-evolving genetic compartments is surely worrisome for plant breeders. This justifies a careful examination of the genomic and biosynthetic potential of FHB-associated Fusaria, which will help us to understand how they may evolve to invade new niches and overcome plant defenses. Given their highly toxic nature and the frequency of predicted production among FHB-associated Fusaria, we believe further studies of APS and APS-producing fungi are warranted - particularly among oat pathogens.

## Materials and methods

### Source of isolates

*Fusarium poae* isolates selected for study were obtained with permission from the research culture collection of Dr. Allen Xue [4] maintained at Agriculture & Agri-Food Canada (Ottawa, ON) (See Additional file 1 for complete list). A subset of these isolates for which genome sequences were deposited in the Canadian Collection of Fungal Cultures (CCFC, Agriculture & Agri-Food Canada, Ottawa, ON). Historic isolates were also sourced from the CCFC (10 Canadian and 10 international). A preliminary assessment of genetic and potential mycotoxin diversity was made by amplification of *TEF1-α* and *TRI* genes (*TRI1* & *TRI*8) (Additional file 2).

### DNA extractions, PCR and Sanger sequence analysis

Single-spore cultures were grown on half-concentration potato dextrose agar (PDA, BD Difco Brand, NJ, USA) plates at 25 °C until nearly confluent. Plugs were transferred to a PDA plate and grown at 25 °C until confluent for genomic DNA extraction and on a synthetic nutrient agar (SNA) plate for 8 days at 25 °C with UV light to prepare frozen glycerol spore stocks.

Fresh mycelium was collected from the PDA plate and placed in a 2 mL screw-cap tube with one ¼” Ceramic Sphere and Lysing Matrix (M.P. Biomedicals). DNA was isolated using the E.Z.N.A. Fungal DNA Mini Kit (Omega Bio-tek Inc.), lysing the tissue in FG1 buffer using a FastPrep24 Sample Preparation System (M.P. Biomedicals). Genomic DNA was eluted in 50 μL Elution Buffer.

*F. poae*-specific primers for *TEF1α*, *TRI1* and *TRI8* were designed based on the published *F. poae* genome [17, 24] (Additional file 15). PCR amplification was performed with the Advantage 2 PCR Polymerase Mix (Takara Bio USA, Inc.) with 200 nM of each primer in a 25 μL reaction volume, and three-step amplification with annealing at 59 °C for 30 cycles. Following amplification, 3 μL was run on a 1% agarose gel. The PCR product was purified using the GenepHlow Gel/PCR kit (Geneaid Biotech Ltd.) as per manufacturer’s instructions and DNA eluted in 30 μL Elution buffer. A 20 ng aliquot of each purified PCR product was sequenced with the forward and reverse primer using a BigDye Terminator v3.1 Cycle Sequencing Kit (Applied Biosystems) as described by the manufacturer in a 10 μL reaction volume with primer concentration of 3.2 μM. After precipitation, reactions were run on an ABI 3500xl Genetic Analyzer (Applied Biosystems). Sequence analysis and alignment was done using the Lasergene12 Core Suite (DNASTAR Inc.) and Geneious R11 (Biomatters Ltd.).

### Fermentation, solvent extraction and UPLC-HRMS analysis

Frozen isolate stocks were thawed and inoculated into slants containing 15 mL of liquid media, where they grew at 25 °C in the dark (3 replicates per isolate per medium). Media conditions included MMK2, CYA, YES, YES with salt-added (“YESIO”), and S2M broth (Additional file 6). After 14 days of incubation, mycelial mats were removed from the liquid media for all conditions except for S2M. For the S2M treatment, isolates were grown at 28 °C in S1M for 4 days, and then transferred to slants containing S2M for 6 days of incubation. In all media conditions, after centrifugation of the supernatants to remove residual mycelia, broth and mycelia were extracted separately in 125 mL Erlenmeyer glass flasks with 15 mL of ethyl acetate for 1 h, shaking at 120 rpm at room temperature. Solvent supernatants were transferred to pre-weighed borosilicate scintillation vials and dried under vaccuum. Extracts were reconstituted in methanol to a concentration of 500 μg/mL and analyzed on a Thermo Ultimate 3000 UPLC coupled to a Thermo LTQ Orbitrap XL high resolution mass spectrometer and a Thermo Dionex Ultimate 3000 Diode array detector (190–800 nm). Chromatography was performed on a Phenomenex C_18_ Kinetex column (50 mm × 2.1 mm ID, 1.7 μm) with a flow rate of 0.35 mL/min, running a gradient of water (+ 0.1% formic acid) and acetonitrile (+ 0.1% formic acid): starting at 5% acetonitrile increasing to 95% acetonitrile by 4.5 mins, held at 95% acetonitrile until 8.0 mins, returning to 5% acetonitrile by 9 mins and held to 10 mins to equilibrate the column to starting conditions. The HRMS was operated in ESI^+^ mode (monitoring a range of 100–2000 *m/z*) using the following parameters: sheath gas [40], auxiliary gas [5], sweep gas [2], spray voltage (4.2 kV), capillary temperature (320 °C), capillary voltage (35 V), and tube lens (100 V).

MS^n^ fragmentation was performed in high resolution on select ions in subsequent experiments using CID at 35 eV. MassWorks™ software (v5.0.0, Cerno Bioscience) was used to improve spectral accuracy and confirm the molecular formulas of annotated ions. The sCLIPS searches were performed in dynamic analysis mode with elements C, H, N, and O allowances set at minimum 1 and maximum 100. Charge was specified as 1 and mass tolerance set to 5 ppm.

Additional experiments were performed to investigate the diversity of apicidin-like signals in isolate extracts via generation of MS^2^ data. This work was performed using a Thermo Q-Exactive Plus mass spectrometer (Thermo Fisher Scientific). See Additional file 16 for details.

### Metabolomics processing and visualization

A detailed explanation of parameters used for metabolomics data processing are provided in Additional file 16. In brief, data preprocessing was carried out using a version of MZMine v2.37 which includes the Ion Identity Networking module [49]. Raw data files were converted into a data matrix of discriminate variables, each being a combination of retention time (RT) and *m/z*. Variables are designated here as mass features, with intensities calculated based on peak area measurements. Data was imported into the R environment, where mass features representing associated adducts and in-source fragments of the same parent ion were grouped using Pearson correlation analysis over a sliding window of elution time. These groupings were compared to data generated during raw data preprocessing via the Ion Identity Networking module which correlated peak shapes of coeluting signals [48]. Mass feature data from the two extraction types (mycelium and broth) were summed, converted to binary form, and then averaged across the five media conditions to form a ‘pseudo-binary’ matrix of detection frequencies for each mass feature.

### Mass feature annotation

Wherever possible, mass features were annotated by comparison of exact *m/z* (< 5 ppm), retention time and MS^2^ fragmentation pattern to commercial standards. 3A-deoxynivalenol, enniatin-A, enniatin-A1, enniatin-B, enniatin-B1 were purchased from Sigma Aldrich (St. Louis, USA). Beauvericin and trichothecene standards 3,15-diacetoxyscirpenol, 15-monoacetoxyscirpenol, neosolaniol, T-2, HT-2, T-2 tetraol, fusarenon-X and nivalenol were purchased from Fermentek (Israel). A 15A-deoxynivalenol standard was purified in-house. Where standards were not available, chemical formulas were predicted based on high resolution exact masses of [M + H]^+^ or [M + Na]^+^ ions (< 5 ppm) combined with isotope abundance patterns (analyzed using MassWorks, Cerno Bioscience). Annotations were supported by analysis of MS^2^ fragmentation patterns using SIRIUS / CSI Finger-ID [50] for in silico predictions and also using the MASST search tool as part of the GNPS workflow [47, 75] for comparison to experimentally derived MS^2^ spectral databases. Additionally, annotations were supported wherever possible by comparison of UV absorbance spectral signatures. To further support the annotation of apicidins, we generated MS^2^ scans of all related signals using a Thermo Q-Exactive mass spectrometer, and performed feature-based molecular network analysis using MZMine2 [49] (special pre-release version 2.37.1corr17.7) and GNPS. Structural hypothesis generation was assisted by the use of mass motif finding using MS2LDA [76].

### Genomic DNA isolation, genome sequencing, assembly and annotation

To generate genomic DNA (gDNA) for both Illumina and Nanopore genome sequencing, spores from *F. poae* isolates (see Additional file 3 for list of isolates) were inoculated in 250 mL Erlenmeyer flasks containing 50 mL first-stage media (Miller and Blackwell, 1986) and incubated at 26 °C, shaking at 170 rpm, for 4 to 5 days. Filtered fungal mycelia were flash frozen and ground in liquid nitrogen with a mortar and pestle until a fine powder was produced. Genomic DNA was extracted using the Illustra Nucleon PhytoPure DNA Extraction Kit (GE Healthcare Bio-Sciences), as per manufacturer’s instructions. The gDNA pellet was reconstituted in 200 μL 10 mM Tris pH 8.0 and the gDNA concentration determined using a FLUOstar OPTIMA fluorometer (BMG LABTECH) and a PicoGreen dsDNA Quantitation Kit (Molecular Probes Inc.). The reconstituted gDNA was mechanically sheared to ~ 300 bp fragments with a Covaris LE220 instrument and used as a template to construct PCR free Libraries with NxSeq AmpFREE Low DNA Library kit (Lucigen) and TruSeq CD dual indices (Illumina) according to the Lucigen’s Library protocol. Indexed libraries were pooled, and sequencing was carried on a NextSeq500/550 (Illumina) using 2 × 150 bp NextSeq High Output Reagent Kit (Illumina) according to the manufacturer’s recommendations in order to obtain paired-end reads. Genome assembly using the generated Illumina data was performed with SPAdes v3.10.1 [77].

For Nanopore long-read gDNA sequencing of *Fp*157, gDNA was isolated from 500 mg of frozen tissue using the Illustra Phytopure Genomic DNA Extraction kit (GE Healthcare). Approximately 6 μg gDNA was then fractionated with a 0.75% agarose gel cassette (10 kb–40 kb) using a SageELF instrument (Sage Science Inc., USA) and the three most abundant fractions were combined for Nanopore sequencing selecting for long reads (SQK-LSK109) using the revC protocol (Oxford Nanopore Technologies, UK) with the following modifications. To reduce loss of DNA after each elution, the initial 70 μL of AMPure XP beads (Beckman Coulter Life Sciences, USA) were reused throughout the procedure. Instead of being transferred to a new tube, the eluted DNA remained in the tube with the AMPure XP beads. Where the addition of new AMPure XP beads was indicated in the protocol, a filter-sterilized 20% PEG 8000/2.5 M solution was added instead. Incubation times throughout the protocol were increased to 10 min. The Long Fragment Buffer (LFB) was used in the adapter ligation step. The library yield was 53 ng. The DNA library was loaded on a FLO-MIN106 flow cell as described in the protocol and run with a MinION device (Oxford Nanopore Technologies, UK) for 48 h.

Genome assembly of *Fp*157 was performed with CANU v1.8 [78] using the sequenced Nanopore reads with default settings with an estimated genome size of 40 Mb (﻿genomeSize = 40 m). The first correction of the CANU assembly was performed using Nanopolish v. 0.11.1 [79]. Illumina reads of *Fp*157 generated from genomic DNA were mapped to the Nanopolish-corrected CANU assembly with BWA v0.7.17 [80] and additional errors were corrected with Pilon v1.23 [81]. Using Geneious, contigs were aligned and compared with those of *F. poae* 2516 (LYXU01) [17] to identify chromosomes 1–4, the mitochondrial contig, and putative ACs of *Fp*157.

To prepare for genome annotation, transcriptome assemblies of *F. poae* 2516 [17] and another isolate, *Fp*133, grown in MMK2 and YES (data not shown), were performed with Trinity v2.8.5 [82] with default settings, from the Illumina sequenced RNA. FUNANNOTATE v1.5.2 [83] was used to annotate the *Fp*157 polished genome using the standard protocol. Gene prediction was performed with the assembled transcriptomes of *F. poae* 2516 and *Fp*133 used as transcript evidence*.* Two predicted genes were manually added to the assembly: *FPOAC1_14145* and *FPOAC1_14146* were annotated based on blastn match to *FpPKS2* and *FpNRPS4* (see Fig. 4).

Following annotation, the command-line version of antiSMASH 5.1 was used to predict the location of biosynthetic gene clusters [34]. Repetitive elements were detected using RepeatMasker and annotated using a merged database consisting of the 2018 version of RepBase [84] and previously annotated repetitive elements from *F. poae* isolate 2516 [56].

PKS and NRPS clade nomenclature used in this publication refers to the published clades from which either the NRPS adenylation domains or the PKS coding regions score highest in blastx comparisons (for example, the *PKS12* cluster in *F. poae* contains a PKS with homology to those in clade 12, which has been associated with an aurofusarin production). Because *Fusarium* terpene synthase nomenclature has not yet been standardized, we adopted terpene synthase clade names associated with studies of *F. langsethiae* and *F. fujikuroi* where applicable [62, 65].

### BUSCO analysis

A total of 4141 single-copy orthologues of house-keeping genes associated with *Hypocreales* (database: Hypocreales_odb10) were identified from Illumina assemblies from nearly all isolates in this study (*Fp*030 was removed due to poor genome sequence quality) including Belgian *F. poae* isolate 2516, using BUSCO v4.0.5 [28]. Nucleotide sequences were aligned using MAFFT v7.470 [85] and trimmed with automated parameter detection using trimal v1.2 [86]. Phylogenetic relationships were inferred using IQTree2.0 [87]. The tree is rooted to the branch containing the outgroup *F. venenatum* (assembly 900,007,375.1, a high quality genome of a close relative to *F. poae* [88]). Evolutionary histories were inferred using the Maximum Likelihood (ML) method and the best model was automatically determined per gene sequence using ModelFinder [89] as part of the IQTree v2.0.6 pipeline [87] utilizing partition modeling to allow genes to evolve under independent models [90]. The tree was calculated using an ultrafast bootstrapping value (*n* = 1000) and drawn to scale, with branch lengths measured in number of substitutions per site [91].

### *APS1* gene survey

To detect *APS1* by PCR, primers were designed and used in a duplex reaction along with *TEF1α* (Additional file 15)*.* To eliminate the possibility of *APS1* false negatives, *TEF1α* was used as a positive control in the duplex reaction to ensure the DNA was amplifiable. All 184 isolates surveyed in this study were tested, as well as 9 Canadian and 10 international (total *n* = 203). PCR was performed as described above except with an annealing temperature of 59 °C. 10 μL was loaded on a 1% agarose gel.

## Supplementary Information

**Additional file 1 **Summary of all *F. poae* isolates screened in this study. Data includes DAOMC accession numbers, crop pathology codes, locations, country of origin, host crop type, groupings inferred by analysis of *TEF1-α, TRI1* and *TRI8* genes, and results from the *APS1* PCR survey.

**Additional file 2 **Jukes-Cantor/Neighbor-joining consensus tree of Clustal Omega alignment of concatenated *TEF1α – TRI1 – TRI8* genomic sequences of 19 representative Ontario and Quebec isolates and one Belgian *F. poae* isolate (genome assembly LYXU01; Vanheule et al. 2016). The isolate name is followed by the number of isolates represented in brackets of the 193 Ontario and Quebec isolates surveyed. The *TEF1α*, *TRI1* and *TRI8* genomic sequences of the 19 isolates have been deposited in Genbank as accession numbers MT571548-MT571566, MT578829-MT578829-MT578847, and MT571567-MT571585, respectively.

**Additional file 3 **Summary of Illumina genome assembly statistics for 37 *F. poae* isolates profiled in this study.

**Additional file 4 **Genome assembly statistics for *Fp*157, assembly WOUF00000000.

**Additional file 5 **LASTZ comparison of *F. poae Fp*157 with *F. poae* 2516 (assembly GCA_001675295.1) and *F. graminearum* PH1 (assembly GCA_000240135.3).

**Additional file 6.** Media conditions used. Formulations for CYA, MMK2, S1M, S2M, YES, YESIO are listed.

**Additional file 7.** Matrix of averaged chemical phenotype data (all mass features included in analysis). Values represent detection frequencies averaged from 5 media conditions. Column headings (mass features) are in RT_*m/z* format, where RT refers to column retention time (minutes) and *m/z* is the mass/charge ratio (< 5 ppm accuracy).

**Additional file 8.** Mirror plots of W-493 A (Top) and W-493 B (Bottom). Upper spectra in each mirror plot represent experimentally derived fragmentation patterns from *F. poae* extracts*,* bottom spectra are from GNPS libraries (spectral matches < 5 ppm are coloured green).

**Additional file 9.** Total ion current and extract ion current chromatographs illustrating butenolide-associated peak (red arrows) eluting during start of run, in region normally sent to waste (blue bracket). Accompanying text explains putative butenolide annotation process.

**Additional file 10.** Mirror plots of apicidin [M + Na] + (top), apicidin B [M + Na] + (middle), and apicidin C [M + Na] + (bottom). Upper spectra in each mirror plot represent experimentally derived fragmentation patterns from *F. poae* extracts*,* bottom spectra are from GNPS libraries (spectral matches < 5 ppm are coloured green).

**Additional file 11.** Mirror plot of MS^2^ spectra from features annotated as apicidin (APS) and APS-G. Overlaid chemical structures are proposed ions associated with specific fragments. Top: spectra of *m/z* [M + H] + = 624.375 (APS structure top right). Spectra coloured purple are present in nearly all spectra associated with apicidins bearing O-methylated tryptophan moiety, including spectra annotated as APS, APS-B, APS-C, and APS-D2. Structures outlined in purple are those proposed to contain indole substructures. Notably, nominal masses 130 and 170 have been associated with tryptophan fragmentation elsewhere^5,6^. Bottom: spectra from parent *m/z* [M + H] + = 555.354 (annotated as APS-G, structure bottom right). Spectra coloured red are present uniquely from MS^2^ scans of this *m/z*, with corresponding proposed ion structures containing phenylalanine outlined in red. Spectra coloured green in bottom plot are *m/z* within 5 ppm match for spectra on top plot.

**Additional file 12.** Expanded molecular network analysis of APS-like spectra with annotations overlaid. Labels are parent ion *m/z*. Node colours indicate ion identities (as identified by IIN module, unless the node outline is grey, in which case the ion was low intensity and didn’t group with informative ions – annotation manual in this case). Blue nodes are [M + H]+, yellow nodes are [M + Na]+, green nodes are [M + NH4]+, purple node is [M-H2O + H]+. Red bordered nodes were annotated as apicidins via in silico spectral analysis, green bordered nodes were annotated as apicidins via GNPS spectral matching (cos > 0.7). Blue lines indicate high spectral matching (cos > 0.7), red lines indicate ion identity matches (peak shape pearson correlation coefficients > 0.8).

**Additional file 13.** Blastn comparison of FpAPS cluster to homologous clusters in *F. sporotrichioides* (PXOF00000000), *F. langsethiae* (JXCE00000000), and *F. incarnatum* (GQ331953).

**Additional file 14.** Duplex PCR screening for presence of *APS1* in *F. poae* genomic DNA. Diagnostic bands for *TEF1α* and *APS1* are indicated by arrows.

**Additional file 15.** List of primer sequences and amplicon sizes for *TEF1α*, *TRI1*, *TRI8* and *APS1*.

**Additional file 16.** Detailed explanation of metabolomics data processing parameters, binary matrix conversion pipeline, qExactive instrument and data processing parameters and mass feature annotation pipeline details.

**Additional file 17.** Compressed folder containing alignments for each of the 4141 orthologs used in BUSCO analysis.

## Data Availability

A representative subset of *Fusarium poae* isolates used for genomic and metabolomics analysis in this study have been made publicly available by request and through standard permissions from the Canadian Collection of Fungal Cultures (Ottawa, ON, Canada; email: aafc.culturesfongiques-daomc-fungalcultures.aac@canada.ca). The annotated genome of *Fp*157 has been deposited with links to BioProject accession number PRJNA578270 in the NCBI BioProject database (https://www.ncbi.nlm.nih.gov/bioproject/ PRJNA578270). Raw reads for *Fp*157 have also been uploaded to NCBI SRA, with accession number SRR13023856 for Illumina reads, and SRR13483968 for Nanopore reads. Sequences used for the BUSCO analysis have been uploaded as a compressed directory of fasta files in Additional file 17.

## Abbreviations

AC: Accessory chromosome
APS: Apicidin
BGC: Biosynthetic gene cluster
BUSCO: Benchmarked universal single copy orthologs
FHB: *Fusarium* head blight
NRPS: Non-ribosomal peptide synthetase
PKS: Polyketide synthase
RIP: Repeat-induced point mutation
STC: Sesquiterpene cyclase
TE: Transposable element
UPLC-HRMS: Ultra-high performance liquid chromatography coupled to high resolution mass spectrometry
