# Supplementary material for: Apicidin biosynthesis is linked to accessory chromosomes in Fusarium poae isolates
Source: BMC Genomics. 2021 Aug 4;22:591. doi: 10.1186/s12864-021-07617-y (PMC8340494; doi:10.1186/s12864-021-07617-y)
Supplement: Supplementary file 2 — Additional file 2 Jukes-Cantor/Neighbor-joining consensus tree of Clustal Omega alignment of concatenated TEF1α – TRI1 – TRI8 genomic sequences of 19 representative Ontario and Quebec isolates and one Belgian F. poae isolate (genome assembly LYXU01; Vanheule et al. 2016). The isolate name is followed by the number of isolates represented in brackets of the 193 Ontario and Quebec isolates surveyed. The TEF1α, TRI1 and TRI8 genomic sequences of the 19 isolates have been deposited in Genbank as accession numbers MT571548-MT571566, MT578829-MT578829-MT578847, and MT571567-MT571585, respectively. [file 12864_2021_7617_MOESM2_ESM.pdf]

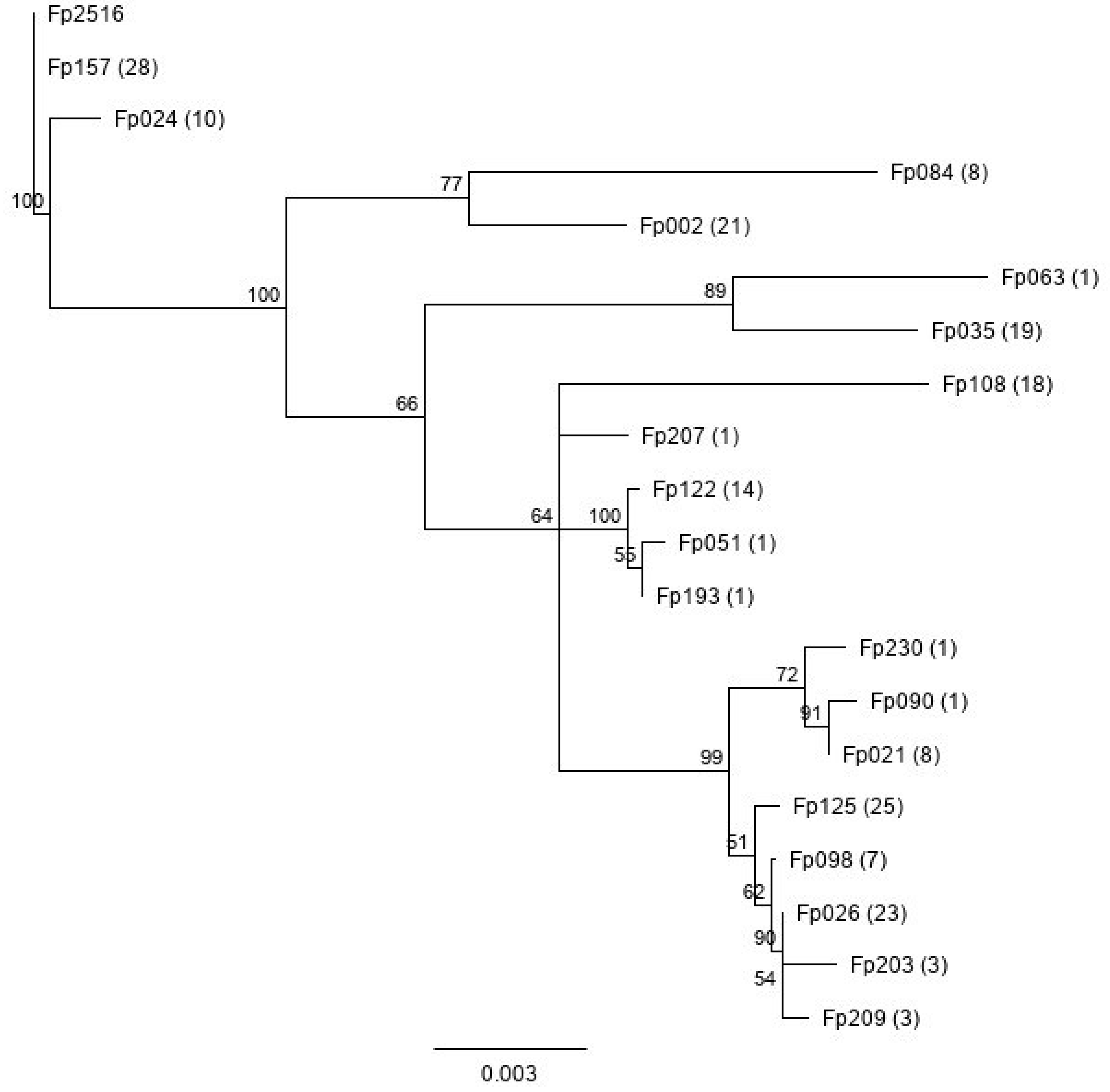


**Additional File 2**. Jukes-Cantor/Neighbor-joining consensus tree of Clustal Omega alignment of concatenated *tef1α – TRI1 – TRI8* genomic sequences of nineteen representative Ontario and Quebec isolates and one Belgian *F. poae* isolate (genome assembly LYXU01; Vanheule *et al.* 2016). The strain name is followed by the number of strains represented in brackets of the 193 Ontario and Quebec strains surveyed. The *TEF1α*, *TRI1* and *TRI8* genomic sequences of the nineteen isolates have been deposited in Genbank as accession numbers MT571548-MT571566, MT578829-MT578829-MT578847, and MT571567-MT571585, respectively.
