# Supplementary material for: Apicidin biosynthesis is linked to accessory chromosomes in Fusarium poae isolates
Source: BMC Genomics. 2021 Aug 4;22:591. doi: 10.1186/s12864-021-07617-y (PMC8340494; doi:10.1186/s12864-021-07617-y)
Supplement: Supplementary file 3 — Additional file 3 Summary of Illumina genome assembly statistics for 37 F. poae isolates profiled in this study. [file 12864_2021_7617_MOESM3_ESM.pdf]

Additional File 3: Illumina genome assembly statistics

Code	DAOMC accession	# scaffolds >1kb	Total length	Coverage (X)	N50	Largest Scaffold	#N's per 100KB	BUSCO% (4494 genes)
Fp001	*252342*	1847	38633878	24	50301	216973	2.18	97.9
Fp013	*252343*	1245	39595310	85	158570	683423	2.12	99.7
Fp016	252208	1556	39034829	40	83192	405430	2.07	99.3
Fp021	252209	2183	38453629	22	35195	192102	2.82	97.1
Fp024	252210	1442	38640026	31	78780	447104	1.81	99.2
Fp026	252211	1452	39158776	56	109452	798962	3.37	99.5
Fp030	252213	6975	38332856	14	7500	134418	8.87	80.7
Fp033	252214	1474	39320325	51	97850	448328	1.41	99.5
Fp034	252215	1336	39268647	53	124392	697434	1.64	99.6
Fp035	252216	1268	39067041	52	123296	487219	2.95	99.5
Fp038	252217	1306	39073284	59	112046	618902	1.3	99.5
Fp039	252218	1594	39074382	37	77961	372157	2.12	99.4
Fp042	252219	1515	39610377	55	102955	986345	2.74	99.6
Fp044	252220	1377	39742799	41	106833	541487	1.76	99.4
Fp049	252221	1520	39336407	48	90632	468692	1.6	99.5
Fp050	252222	1411	39498519	45	101750	883907	2.03	99.5
Fp051	252223	1363	38905541	53	101177	881013	1.49	99.5
Fp059	252224	1395	39348608	52	103044	505886	1.99	99.6
Fp063	252225	1267	39115705	84	119107	586963	1.15	99.6
Fp065	252226	1328	39593045	73	124859	939576	1.04	99.7
Fp066	252227	1372	38876433	62	108785	577974	1.44	99.5
Fp072	252228	1182	39617292	77	169579	552873	1.97	99.6
Fp073	252229	1458	39478102	42	103732	573944	2.86	99.5
Fp076	252230	1288	38966292	51	118388	704647	2.24	99.6
Fp078	252231	1447	39228968	56	96802	520420	2.35	99.4
Fp081	252232	1473	39343540	35	90005	765074	1.78	99.4
Fp084	252233	1594	38805961	32	69039	671399	1.19	99.2
Fp086	252234	2028	39081031	25	46167	228412	2.82	97.9
Fp105	252236	1279	39846319	69	133456	941253	2.43	99.7
Fp108	252237	1338	39501979	65	127759	854759	1.85	99.7
Fp118	252238	1319	39699314	95	130281	1006554	1.55	99.6
Fp122	252239	1290	39790632	78	141127	1062973	2.13	99.6
Fp125	252240	1235	39779437	80	126879	649987	2.26	99.6
Fp144	252241	1284	39640194	92	128957	703960	2.11	99.6
Fp146	252242	1518	38551751	35	78420	322490	0.93	99.5
Fp155	252243	1342	40066615	94	129972	991315	1.4	99.6
Fp157	252244	1184	39327233	121	154309	877588	1.96	99.7
Mean		1580.68	39254191.3	56	104393.2	629782.2	2.15	
Median		1375						
