# Supplementary material for: Apicidin biosynthesis is linked to accessory chromosomes in Fusarium poae isolates
Source: BMC Genomics. 2021 Aug 4;22:591. doi: 10.1186/s12864-021-07617-y (PMC8340494; doi:10.1186/s12864-021-07617-y)
Supplement: Supplementary file 4 — Additional file 4 Genome assembly statistics for Fp157, assembly WOUF00000000. [file 12864_2021_7617_MOESM4_ESM.pdf]

Additional File 4: Fp157 genome assembly of Nanopore reads using Canu, Nanopolished with Nanopore and Illumina reads.

Name	Length	GC Content	Predicted genes (#)	Notes	Genes/Mb	Repeat content (%)	RIP (%)
Chr1	12123814	46.60%	3935	Core	324.5678299	1.92	3.9
Chr2	9989830	46.20%	3421	Core	342.4482699	2.85	3.5
Chr3	8051089	46.30%	2778	Core	345.04649	2.43	3.9
Chr4	8366728	46.70%	2704	Core	323.1848818	1.93	3.1
Contig_1	1877593	47.90%	480	SNC	255.646458	10.77	0.2
Contig_2	1457426	48.70%	323	SNC	221.6236022	11.57	0.7
Contig_3	865838	46.70%	235	SNC	271.4133591	10.12	0.2
Contig_4	578794	46.80%	134	SNC	231.5158761	13.24	0.6
Contig_5	140862	31.50%	-	mitochondrial	-	1.9	-
Contig_6	169837	49.00%	36	SNC	211.9679457	13.23	0
Contig_7	155888	48.20%	40	SNC	256.5944781	13.84	0
Contig_8	122757	51.40%	-	rDNA	-	68.82	-
Contig_9	100057	50.20%	28	SNC	279.8404909	19.29	1

Nanopore stats	Fp157
# reads (passed filtering)	340,123
longest read	190,733
median length	16,339
mean length	21,465
mean qscore	10.24
