# Supplementary material for: Apicidin biosynthesis is linked to accessory chromosomes in Fusarium poae isolates
Source: BMC Genomics. 2021 Aug 4;22:591. doi: 10.1186/s12864-021-07617-y (PMC8340494; doi:10.1186/s12864-021-07617-y)
Supplement: Supplementary file 5 — Additional file 5 LASTZ comparison of F. poae Fp157 with F. poae 2516 (assembly GCA_001675295.1) and F. graminearum PH1 (assembly GCA_000240135.3). [file 12864_2021_7617_MOESM5_ESM.pdf]

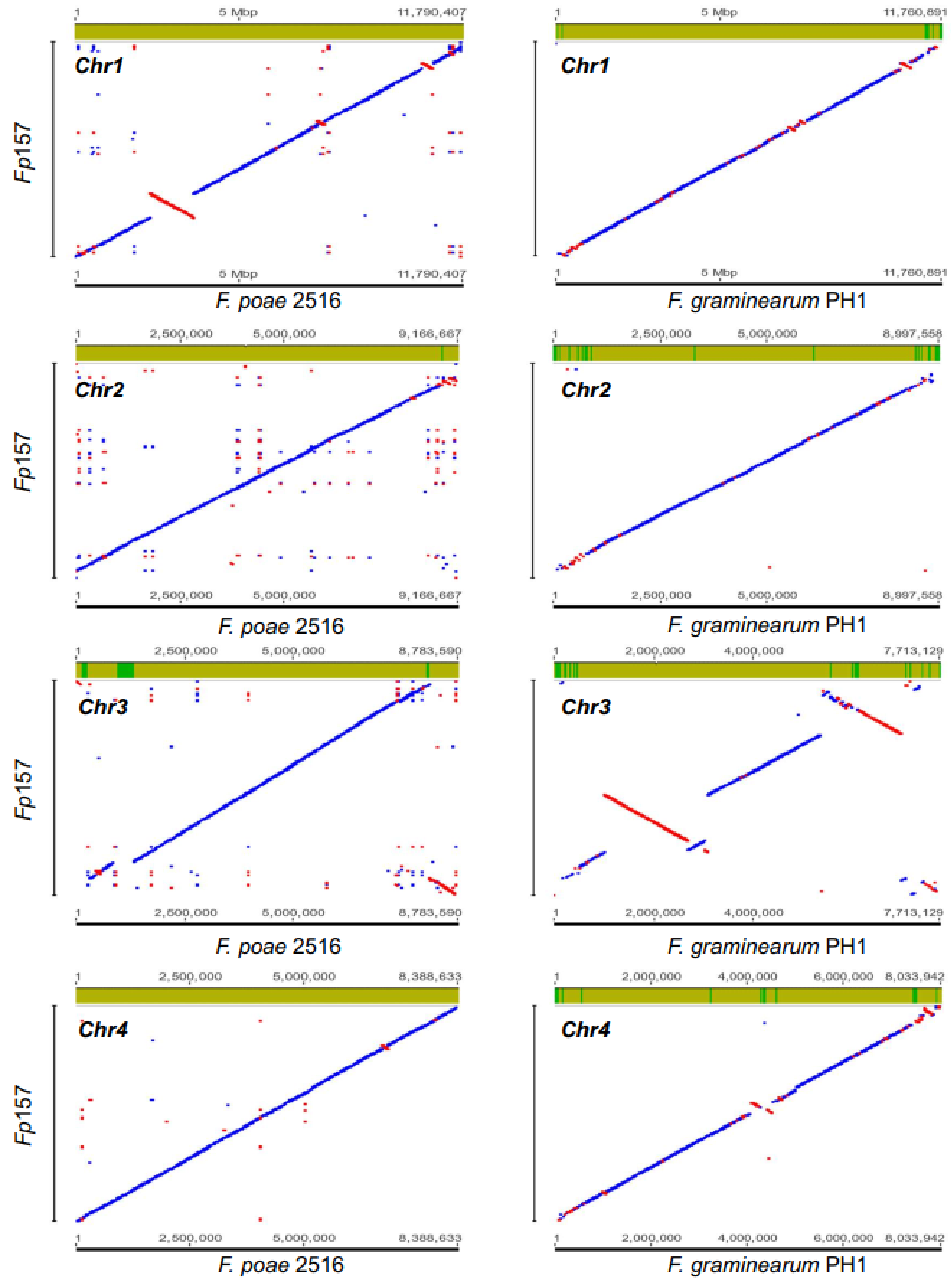

Additional File 5: LASTZ comparison of *F. poae Fp*157 with *F. poae* 2516 (assembly GCA_001675295.1) and *F. graminearum* PH1 (assembly GCA_000240135.3).
