# Supplementary material for: Apicidin biosynthesis is linked to accessory chromosomes in Fusarium poae isolates
Source: BMC Genomics. 2021 Aug 4;22:591. doi: 10.1186/s12864-021-07617-y (PMC8340494; doi:10.1186/s12864-021-07617-y)
Supplement: Supplementary file 6 — Additional file 6. Media conditions used. Formulations for CYA, MMK2, S1M, S2M, YES, YESIO are listed. [file 12864_2021_7617_MOESM6_ESM.pdf]

Additional File 6: Media ingredients

CYA: (Czapek Yeast (Autolysate) extract)

**Liquid 1L**			**Trace Elements 100 mL**		
3	g	$NaNO_3$	1	g	$ZnSO_4.7H_2O$
1	g	KH_2PO_4	0.5	g	$CuSO_4.5H_2O$
500	mg	KCl	100	mL	Distilled Water
500	mg	$MgSO_4.7H_2O$			
10	mg	$FeSO_4.7H_2O$			
5	g	Yeast extract			
30	g	Sucrose			
1000	mL	Distilled Water			
1000	uL	Trace Elements			

MMK2:

Liquid 1 L

40	g	Mannitol
5	g	Yeast Extract
4.3	g	Murashuge & Skoog salts
1000	mL	Distilled Water

YES: (Yeast Extract Sucrose)

Liquid 1L

20	g	Yeast extract
150	g	Sucrose
500	mg	$MgSO_4.7H_2O$
1000	mL	Distilled Water

YESIO:

YESIO is YES with salt stress. To YES media formulation above, add:

1L

18	g	Instant Ocean

1st Stage - 2nd Stage Media for high DON production (Harris lab)

1st Stage media (S1M)

__Liquid__
1L

3	g	NH_4Cl
2	g	$MgSO_4.7H_2O$
0.2	g	$FeSO_4.7H_2O$
2	g	KH_2PO_4
2	g	peptone
2	g	yeast extract
2	g	malt extract
20	g	glucose

2nd Stage Media (S2M)
__Liquid__
1L

1	g	$(NH_4)_2HPO_4$
3	g	KH_2PO_4
0.2	g	$MgSO_4.7H_2O$
5	g	NaCl
40	g	sucrose
10	g	glycerol

Synthetic Nutrient Agar (SNA)
__Agar (solid)__
1L

1	g	KH_2PO_4
1	g	KNO_3
0.5	g	$MgSO_4.7H_2O$
0.5	g	KCl
0.2	g	Glucose
0.2	g	Sucrose
20	g	Agar
1	L	Distilled water
