# Supplementary material for: Apicidin biosynthesis is linked to accessory chromosomes in Fusarium poae isolates
Source: BMC Genomics. 2021 Aug 4;22:591. doi: 10.1186/s12864-021-07617-y (PMC8340494; doi:10.1186/s12864-021-07617-y)
Supplement: Supplementary file 9 — Additional file 9. Total ion current and extract ion current chromatographs illustrating butenolide-associated peak (red arrows) eluting during start of run, in region normally sent to waste (blue bracket). Accompanying text explains putative butenolide annotation process. [file 12864_2021_7617_MOESM9_ESM.pdf]

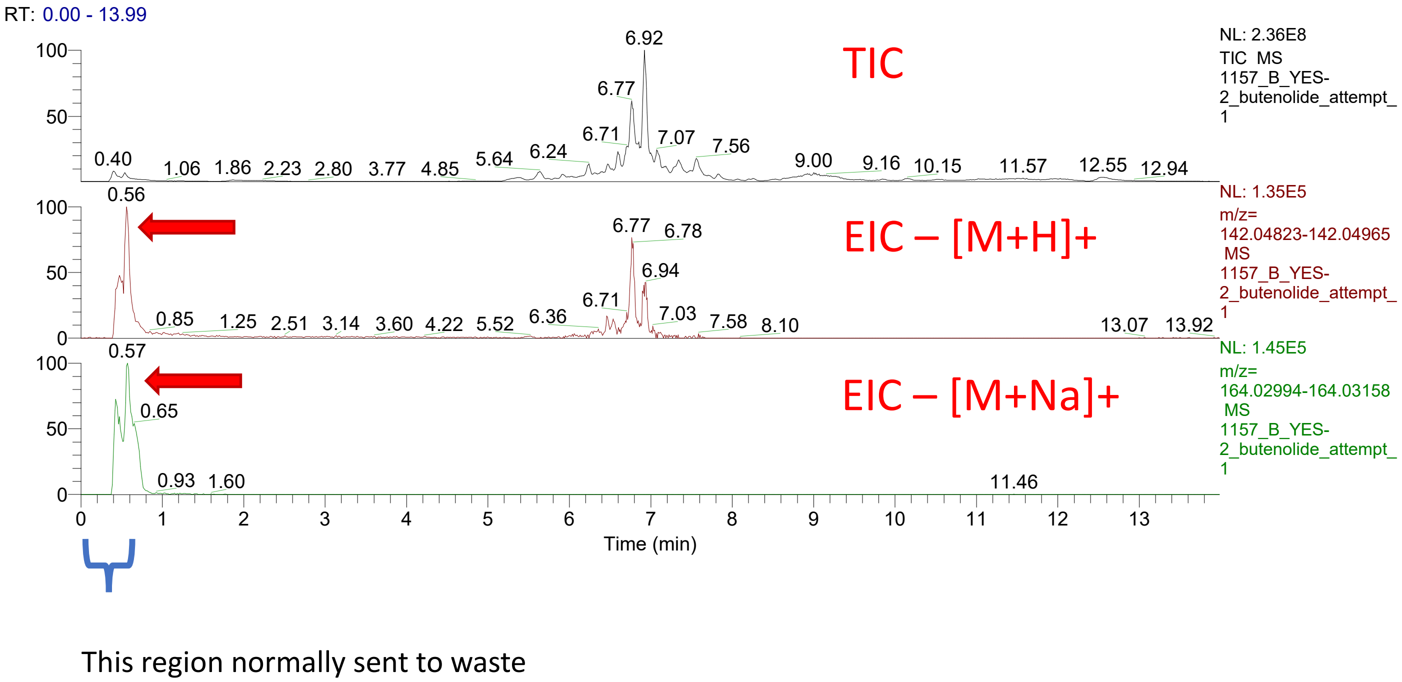

Additional File 9. Total ion current and extract ion current chromatographs illustrating butenolide-associated peak (red arrows) eluting during start of run, in region normally sent to waste (blue bracket).

Butenolide identification rationale and adjusted protocol

We reasoned that butenolide, being a small, polar molecule, was likely being diverted to waste in our LCMS protocol, as it would elute from the column along with a solvent front we did not want to process. We therefore processed some extracts of *Fp*157 using a slightly modified protocol to the one described here, where the initial 0.5 minutes of elution wasn't sent to waste, and we were then able to detect butenolide-associated signals in the extracts of Fp157. In the absence of a commercial standard for butenolide, MS^2 fragmentation spectra of the putative [M+H]+ ion was analyzed using *in silico* fragmentation spectral analysis, and while butenolide was not ranked as the top hit using CSI FINGER-ID, it was ranked as the top hit from a fungal source using MSFINDER v3.44, another *in silico* fragment prediction tool.
