# Supplementary material for: Apicidin biosynthesis is linked to accessory chromosomes in Fusarium poae isolates
Source: BMC Genomics. 2021 Aug 4;22:591. doi: 10.1186/s12864-021-07617-y (PMC8340494; doi:10.1186/s12864-021-07617-y)
Supplement: Supplementary file 10 — Additional file 10. Mirror plots of apicidin [M + Na] + (top), apicidin B [M + Na] + (middle), and apicidin C [M + Na] + (bottom). Upper spectra in each mirror plot represent experimentally derived fragmentation patterns from F. poae extracts, bottom spectra are from GNPS libraries (spectral matches < 5 ppm are coloured green). [file 12864_2021_7617_MOESM10_ESM.pdf]

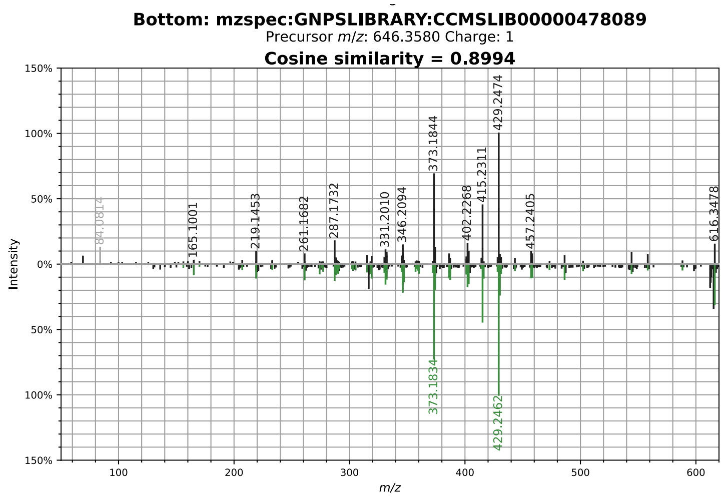

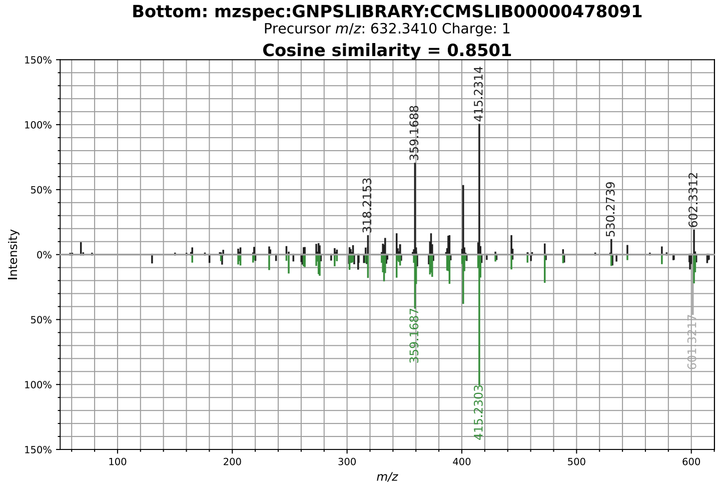

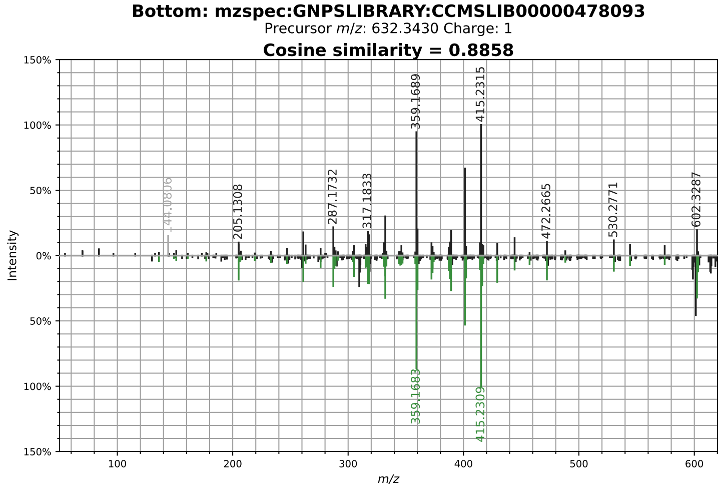

Additional File 10. Mirror plots of apicidin [M+Na]+ (top), apicidin B [M+Na]+ (middle), and apicidin C [M+Na]+ (bottom). Upper spectra in each mirror plot (black lines) represent experimentally derived fragmentation patterns from *F. poae* extracts, bottom spectra are from GNPS libraries (matches <5ppm in green).
