# Supplementary material for: Apicidin biosynthesis is linked to accessory chromosomes in Fusarium poae isolates
Source: BMC Genomics. 2021 Aug 4;22:591. doi: 10.1186/s12864-021-07617-y (PMC8340494; doi:10.1186/s12864-021-07617-y)
Supplement: Supplementary file 11 — Additional file 11. Mirror plot of MS2 spectra from features annotated as apicidin (APS) and APS-G. Overlaid chemical structures are proposed ions associated with specific fragments. Top: spectra of m/z [M + H] + = 624.375 (APS structure top right). Spectra coloured purple are present in nearly all spectra associated with apicidins bearing O-methylated tryptophan moiety, including spectra annotated as APS, APS-B, APS-C, and APS-D2. Structures outlined in purple are those proposed to contain indole substructures. Notably, nominal masses 130 and 170 have been associated with tryptophan fragmentation elsewhere5,6. Bottom: spectra from parent m/z [M + H] + = 555.354 (annotated as APS-G, structure bottom right). Spectra coloured red are present uniquely from MS2 scans of this m/z, with corresponding proposed ion structures containing phenylalanine outlined in red. Spectra coloured green in bottom plot are m/z within 5 ppm match for spectra on top plot. [file 12864_2021_7617_MOESM11_ESM.pdf]

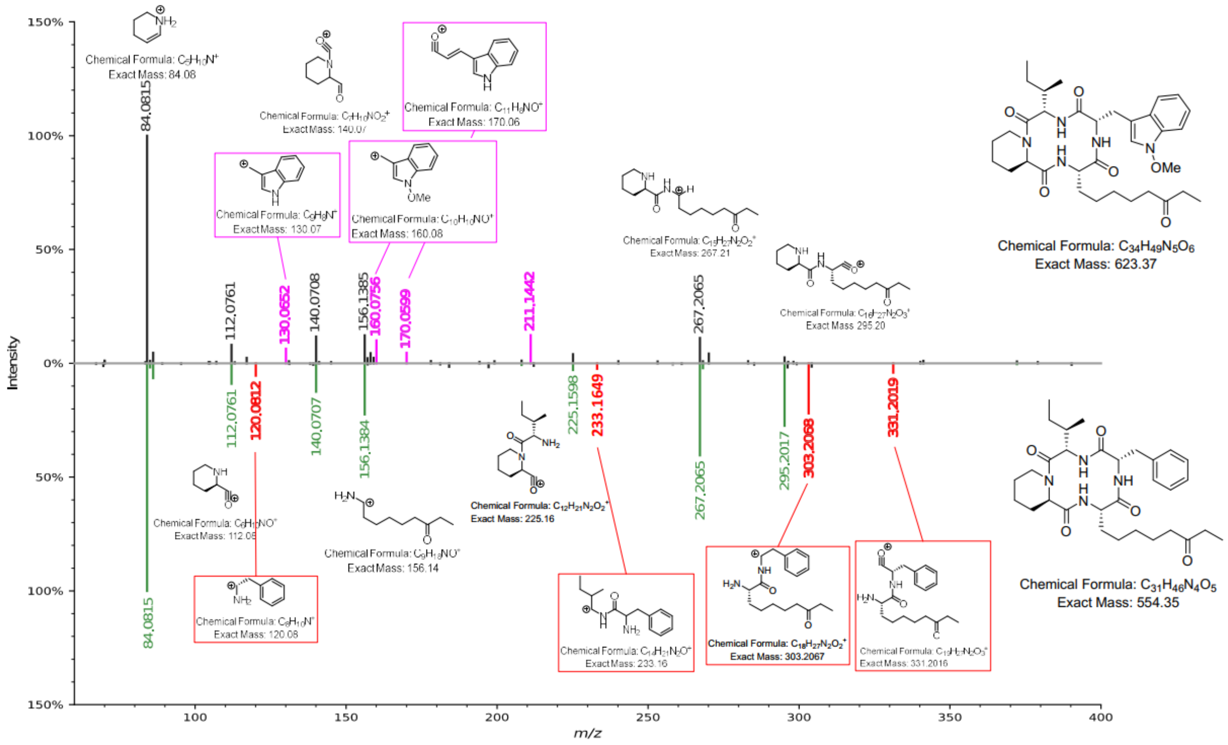

Additional File 11: Mirror plot of MS^2 spectra from features annotated as apicidin (APS) and APS-G. Overlaid chemical structures are proposed ions associated with specific fragments. Top: spectra of *m/z* [M+H]+ = 624.375 (APS structure top right). Spectra coloured purple are present in nearly all spectra associated with apicidins bearing O-methylated tryptophan moiety, including spectra annotated as APS, APS-B, APS-C, and APS-D2. Structures outlined in purple are those proposed to contain indole substructures. Notably, nominal masses 130 and 170 have been associated with tryptophan fragmentation elsewhere[5,6]. Bottom: spectra from parent *m/z* [M+H]+ = 555.354 (annotated as APS-G, structure bottom right). Spectra coloured red are present uniquely from MS^2 scans of this *m/z*, with corresponding proposed ion structures containing phenylalanine outlined in red. Spectra coloured green in bottom plot are *m/z* within 5ppm match for spectra on top plot.
