# Supplementary material for: Apicidin biosynthesis is linked to accessory chromosomes in Fusarium poae isolates
Source: BMC Genomics. 2021 Aug 4;22:591. doi: 10.1186/s12864-021-07617-y (PMC8340494; doi:10.1186/s12864-021-07617-y)
Supplement: Supplementary file 12 — Additional file 12. Expanded molecular network analysis of APS-like spectra with annotations overlaid. Labels are parent ion m/z. Node colours indicate ion identities (as identified by IIN module, unless the node outline is grey, in which case the ion was low intensity and didn’t group with informative ions – annotation manual in this case). Blue nodes are [M + H]+, yellow nodes are [M + Na]+, green nodes are [M + NH4]+, purple node is [M-H2O + H]+. Red bordered nodes were annotated as apicidins via in silico spectral analysis, green bordered nodes were annotated as apicidins via GNPS spectral matching (cos > 0.7). Blue lines indicate high spectral matching (cos > 0.7), red lines indicate ion identity matches (peak shape pearson correlation coefficients > 0.8). [file 12864_2021_7617_MOESM12_ESM.pdf]

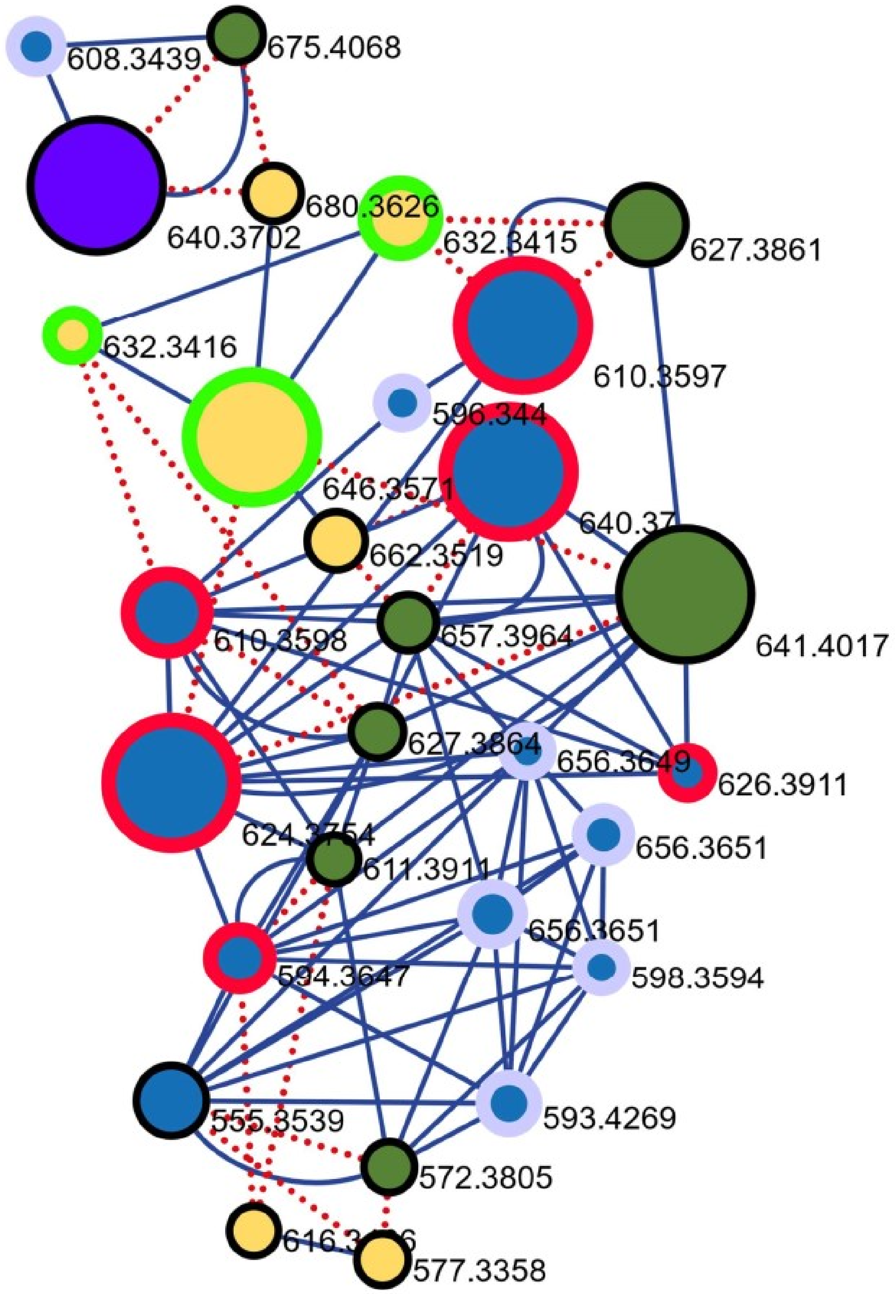

Additional File 12: Expanded molecular network analysis of APS-like spectra with annotations overlaid. Labels are parent ion *m/z*. Node colours indicate ion identities (as identified by IIN module, unless the node outline is grey, in which case the ion was low intensity and didn't group with informative ions – annotation manual in this case). Blue nodes are [M+H]+, yellow nodes are [M+Na]+, green nodes are [M+NH4]+, purple node is [M-H2O+H]+. Red bordered nodes were annotated as apicidins via *in silico* spectral analysis, green bordered nodes were annotated as apicidins via GNPS spectral matching (cos >0.7). Blue lines indicate high spectral matching (cos >0.7), red lines indicate ion identity matches (peak shape pearson correlation coefficients > 0.8).
