# Supplementary material for: Apicidin biosynthesis is linked to accessory chromosomes in Fusarium poae isolates
Source: BMC Genomics. 2021 Aug 4;22:591. doi: 10.1186/s12864-021-07617-y (PMC8340494; doi:10.1186/s12864-021-07617-y)
Supplement: Supplementary file 13 — Additional file 13. Blastn comparison of FpAPS cluster to homologous clusters in F. sporotrichioides (PXOF00000000), F. langsethiae (JXCE00000000), and F. incarnatum (GQ331953). [file 12864_2021_7617_MOESM13_ESM.pdf]

Additional File 13: Blastn comparison of FpAPS cluster to homologous clusters in F. sporotrichioides (PXOF00000000), F. langsethiae (JXCE00000000), and F. incarnatum (GQ331953).

Name	*Fp* gene ID	Size (aa)	***F.sporo*** **PXOF00000000 Blastn (ID%/Cov%)**	***F. lang*** **JXCE00000000 Blastn (ID%/Cov%)**	***F.incarnatum GQ331953**** **Blastn (ID%/Cov%)**	Function
APS1	FUN_013755	5106	98/100	98/100	89.2/100	Non-ribosomal peptide synthase
APS2	FUN_013744	366	87.6/100	88.2/100	81.7/99.8	Transcription factor
APS3	FUN_013745	306	86.4/100	86.2/100	81.5/100	Pyrroline reductase
APS4	FUN_013746	360				Aminotransferase
APS5	FUN_013747	1615	98.8/100	98.2/100	86.6/100	Fatty acid synthase
APS6	FUN_013749	391	98.2/100	98.1/100	87.2/100	O-Methyl transferase
APS7	FUN_013750	534	99/100	99.2/100	89.2/99.8	Cytochrome P450
APS8	FUN_013751	512	98/100	98.2/100	87.2/99.8	Cytochrome P450
APS9	FUN_013752	590	96.7/100	96.4/100	87.6/94.4	FAD-dependent oxidase
APS10	FUN_013753	251	98.5/100	97.6/100	91.6/100	Short-chain reductase
APS11	FUN_013754	571	98.5/100	97.9/100	88.9/99.6	Efflux pump
APS12	FUN_013748	845	98.2/100	98.2/100	86.8/100	CYT b5-like reductase

*From Jin *et al*. 2010
