# Supplementary material for: Apicidin biosynthesis is linked to accessory chromosomes in Fusarium poae isolates
Source: BMC Genomics. 2021 Aug 4;22:591. doi: 10.1186/s12864-021-07617-y (PMC8340494; doi:10.1186/s12864-021-07617-y)

Additional File 14. Representative subset of strains screened using duplex PCR screening for presence of *APS1* in *F. poae* genomic DNA. Diagnostic bands for *TEF1a* and *APS1* are indicated by arrows. *Fpa*1 and *Fpa*2 were not included in this study.

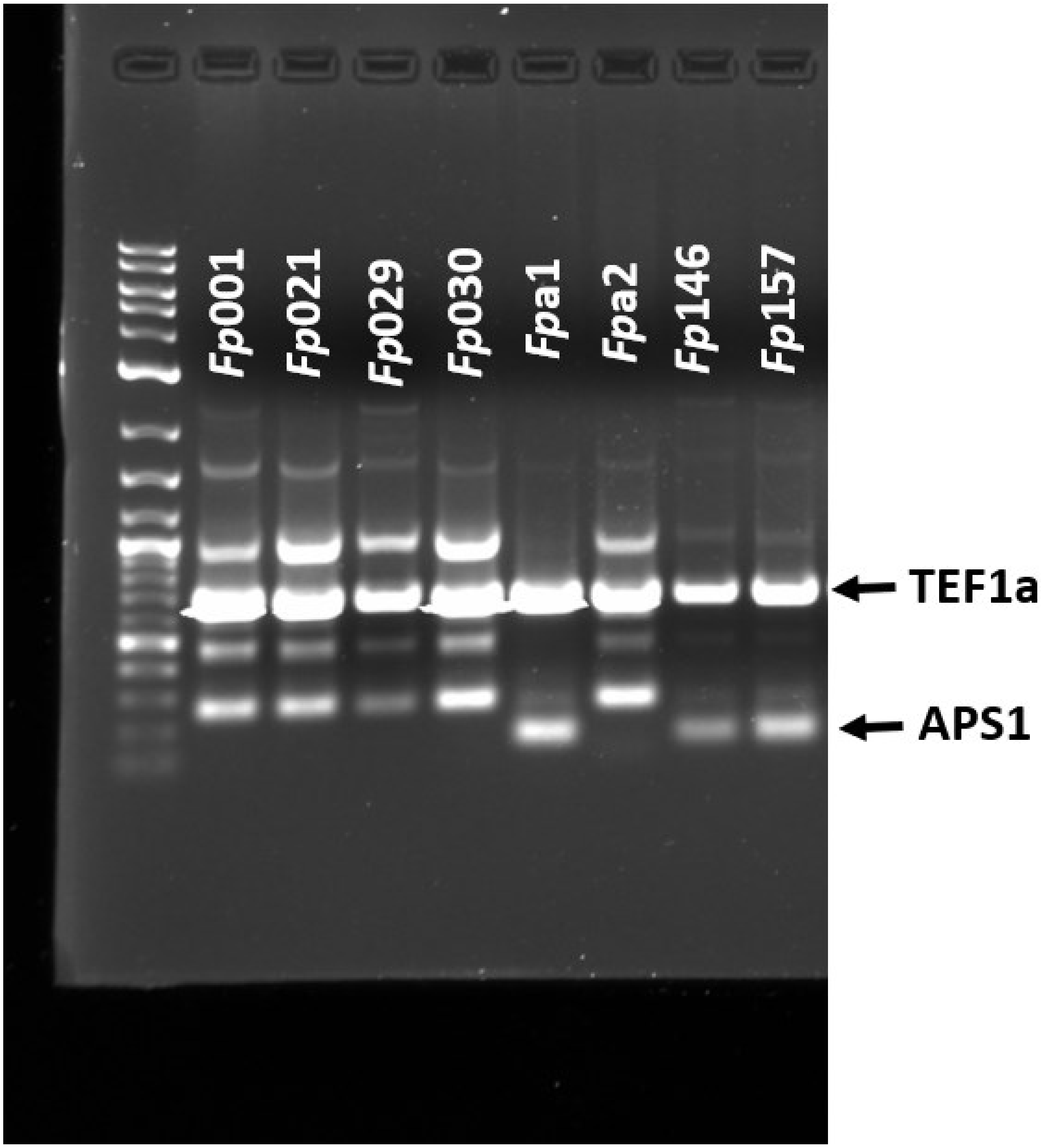

Supplement: Supplementary file 14 — Additional file 14. Duplex PCR screening for presence of APS1 in F. poae genomic DNA. Diagnostic bands for TEF1α and APS1 are indicated by arrows. [file 12864_2021_7617_MOESM14_ESM.pdf]
