# Supplementary material for: Apicidin biosynthesis is linked to accessory chromosomes in Fusarium poae isolates
Source: BMC Genomics. 2021 Aug 4;22:591. doi: 10.1186/s12864-021-07617-y (PMC8340494; doi:10.1186/s12864-021-07617-y)
Supplement: Supplementary file 15 — Additional file 15. List of primer sequences and amplicon sizes for TEF1α, TRI1, TRI8 and APS1. [file 12864_2021_7617_MOESM15_ESM.pdf]

Additional File 15. List of primer sequences and amplicon sizes.

Primer name	Primer sequence	Amplicon size (bp)
TEF1α fwd:	5'-ATGGGTAAGGAGGAGAAGACT-3'	682
TEF1α rev:	5'-GGAAGTACCAGTGATCATGTT-3'	
TRI1 fwd:	5'-CGGGCCTGTGGACATCT-3'	725
TRI1 rev:	5'-GGGTTTCTTGAGCCAATGGAAT-3'	
TRI8 fwd:	5'-ACAACATCCTCTATCGCACAAC-3'	802
TRI8 rev:	5'-GTGATATCCCATGATGCCTTCC-3'	
APS1 fwd:	5'-GAAGCAGACATTGGCAACCG-3'	150
APS1 rev:	5'-GAAGAGTCCGATAGCAGGGC-3'	
