# Supplementary material for: Apicidin biosynthesis is linked to accessory chromosomes in Fusarium poae isolates
Source: BMC Genomics. 2021 Aug 4;22:591. doi: 10.1186/s12864-021-07617-y (PMC8340494; doi:10.1186/s12864-021-07617-y)
Supplement: Supplementary file 16 — Additional file 16. Detailed explanation of metabolomics data processing parameters, binary matrix conversion pipeline, qExactive instrument and data processing parameters and mass feature annotation pipeline details. [file 12864_2021_7617_MOESM16_ESM.pdf]

Additional File 16: Detailed explanation of metabolomics data processing parameters, binary matrix conversion pipeline, qExactive instrument and data processing parameters and mass feature annotation pipeline details.

Metabolomics Data Preprocessing

Data preprocessing was carried out using MZMine v2.51[1]. Raw data files included methanol blanks run after every sixth sample, and all were carefully examined to determine a minimum noise level threshold for data analysis. Masses were detected with a noise threshold set to 1.0 E^4 . The ADAP algorithm was used for chromatogram building with a minimum group size of 5, group intensity threshold of 1.0E^5, and minimum highest intensity threshold set to 5.0 E^6. All *m/z* tolerance settings were set to 0.01 *m/z* or 5.0 ppm, and all RT tolerance settings were set to 0.05 min. Chromatograms were then deconvoluted using the ADAP wavelets algorithm with a signal to noise threshold of 5, minimum feature height of 1.0E^6, co-efficient area threshold of 110, peak duration range of 0 - 10, and wavelet RT range of 0 - 0.10. These settings were determined by examination of their effects on previewed ion chromatographs within MZMine. The data was then cleared of isotopes, aligned, and converted into a data matrix of discriminate variables (a combination of RT and *m/z* – designated here as mass features) based on peak area measurements. Peaks were aligned between samples using the Join Alignment function with a 2:1 weight for *m/z* vs. RT. Gaps in the data set where mass features fell below the noise limit detection threshold were backfilled using the PeakFinder gap-filling algorithm. Finally, peak area values were normalized to the total ion current for each sample.

Data conversion to binary matrix

The resulting data matrix of mass features was exported from MZMine and imported into the R environment, where signals consistent to methanol blanks, media blanks and reserpine QC standard were removed using an in-house script. Likely adducts and in-source fragments were grouped using Pearson correlation analysis over a sliding window of elution time. Mass features were grouped if their intensities correlated across samples (correlation coefficients threshold set to 0.85) and if they eluted from the column within 0.02 minutes of each other. The correlated groups were then assigned a representative ion by manual determination of the most likely [M+H]+ or [M+Na]+ ion. This process was assisted by a recent release of MZMine 2.37 containing an Ion Identity Networking module, used to correlate peak shapes of coeluting signals to suggest their relationship as either adducts or in-source fragments of the same parent ion[2].

In developing the binary matrices, errors likely introduced at this stage by the gap-filling ‘PeakFinder’ algorithm were reduced by clearing all values below a threshold of 10. This arbitrary threshold was identified by close examination of a histogram of the lower-end values of the data set in comparison to extracted ion chromatographs of raw data. Sample data from the two extraction types (mycelium and broth) were summed together per replicate, and the data was then converted to binary form. Mass features were filtered out if they occurred in at less than 2 of the 3 replicates (ie <0.65). The data

was made binary again and averaged across the five media conditions to form a ‘pseudo-binary’ matrix of detection frequencies for each feature.

For the generation of the chemical phenotype heatmap (**Figure 1**), row dendrograms are calculated from “ward.D2” clustering of Euclidean distance matrix and are not indicative of phylogenetic relationships. Columns represent mass features. Column dendrograms were computed as an hclust object using “average” linkage clustering of Euclidean distances, and then seriated using the “GW” parameter of the “seriation” R package. The heatmap was generated using the “ComplexHeatmap” R package.

qExactive Data Processing

Dried extracts were resuspended with 50 mL in 50:50 acetonitrile:water with 0.1% formic acid for LC/MS analysis. All samples were filtered through 0.2 um PTFE membrane filters. All samples were analyzed by nanoLC coupled to the Q-Exactive Plus mass spectrometer (Thermo Fisher Scientific). Chromatographic separation of metabolites was performed on a Proxeon EASY nLC II System (Thermo Fisher Scientific) equipped with a Thermo Scientific™ Acclaim™ PepMap™ RSLC C18 column (P/N ES800A), 15 cm x 75 µm ID, 3 µm, 100 Å employing a water/acetonitrile/0.1% formic acid gradient. Samples were loaded onto the column for 60 min at a flow rate of 0.25 µl/min. Compounds were separated using a linear gradient from 10 to 100% of acetonitrile for 45 min, followed by washing 5 min at 100% of acetonitrile, then using a gradient from 100 to 10% of acetonitrile for 5 min and washing for 5 min at 100% of water. Eluted compounds were directly sprayed into mass spectrometer using positive electrospray ionization (ESI) at an ion source temperature of 250°C and an ionspray (Thermo Scientific™ EASY spray) voltage of 2.1 kV. The FTMS scan type was full MS/data dependent (dd)-MS^2. The parameters of the full mass scan were as follows: a resolution of 70,000, an auto gain control target under 3×10^6, a maximum isolation time of 100 ms, and an m/z range of 100–1500. The parameters of the dd-MS^2 scan were as follows: a resolution of 17,500, an auto gain control target under 1×10^5, a maximum isolation time of 100 ms, a loop count of top 10 peaks, an isolation window of m/z 2, a normalized collision energy of 35 and dynamic exclusion duration of 10 s,. The LC-FTMS system was controlled using Xcalibur 4 software (Thermo Fisher Scientific), and data were collected with the same software.

MS^2 scans linked to mass features were networked using feature-based molecular network analysis. Data preprocessing was performed using MZMine2[1] (special pre-release version 2.37.1corr17.7) utilizing the ion identity networking (IIN) module[2] Features were filtered from the analysis if they had less than 3 isotopes per feature, or if they had neither MS^2 scan nor ion identity annotation. MS^2 spectra were linked in the network if they had at least 6 shared fragments and a cosine score greater than 0.7. Ion identities (adducts or in-source fragments) were grouped using Pearson correlation analysis of peak shapes with

Mass feature annotation

Wherever possible, mass features were annotated by comparison of exact *m/z* (<5ppm), retention time and MS^2 fragmentation pattern to commercial standards. 3A-deoxynivalenol, enniatin-A, enniatin -A1, enniatin -B, enniatin -B1 were purchased from Sigma Aldrich (St. Louis, USA). Beauvericin and trichothecene standards 3,15-DAS, 15-MAS, NEO, T-2, HT-2, T-2 tetraol, fusarenon-X and nivalenol were purchased from Fermentek (Israel). A 15A-deoxynivalenol standard was purified in-house. Where standards were not available, annotations were made based on exact masses of $[M+H]^+$ or $[M+Na]^+$ ions (<5ppm), chemical formulas predicted via high resolution exact mass measurements combined with isotope abundance patterns (analyzed using MassWorks, Cerno Bioscience), MS^2 fragmentation patterns compared to *in silico* predictions using SIRIUS / CSI Finger-ID, and lastly by comparison of MS^2 data to experimentally derived MS^2 data of known compounds using the MASST search tool as part of the GNPS workflow. Annotations were supported in the case of rubrofusarin, aurofusarin and fusarin-like mass features by comparison of UV absorbance spectra. To further support the annotation of apicidins, we generated MS^2 scans of all related signals using a Thermo Q-Exactive mass spectrometer, and performed feature-based molecular network analysis using MZMine2 (special pre-release version 2.37.1corr17.7) utilizing the ion identity networking (IIN) module in addition to *in silico*-based fragmentation predictions described above. Structural hypothesis generation was assisted by the use of mass motif finding using MS2LDA.
